# Advanced waveform analysis of the photoplethysmogram signal using complementary signal processing techniques for the extraction of biomarkers of cardiovascular function

**DOI:** 10.1177/20480040231225384

**Published:** 2024-02-01

**Authors:** Aristide Jun Wen Mathieu, Miquel Serna Pascual, Peter H Charlton, Maria Volovaya, Jenny Venton, Philip J Aston, Manasi Nandi, Jordi Alastruey

**Affiliations:** 1 Department of Biomedical Engineering, School of Biomedical Engineering and Imaging Sciences, Faculty of Life Sciences and Medicine, 4616 King’s College London, St Thomas’ Hospital, London, UK; 2 School of Cancer and Pharmaceutical Science, Faculty of Life Sciences and Medicine, King's College London, London, UK; 3 Department of Public Health and Primary Care, 2152 University of Cambridge, Cambridge, Cambridgeshire, UK; 4 Department of Mathematics, 3660 University of Surrey, Guildford, UK

**Keywords:** Photoplethysmogram, fiducial point analysis, Symmetric Projection Attractor Reconstruction, pulse wave morphology, pulse wave variability, vascular aging

## Abstract

**Introduction:**

Photoplethysmogram signals from wearable devices typically measure heart rate and blood oxygen saturation, but contain a wealth of additional information about the cardiovascular system. In this study, we compared two signal-processing techniques: fiducial point analysis and Symmetric Projection Attractor Reconstruction, on their ability to extract new cardiovascular information from a photoplethysmogram signal. The aim was to identify fiducial point analysis and Symmetric Projection Attractor Reconstruction indices that could classify photoplethysmogram signals, according to age, sex and physical activity.

**Methods:**

Three datasets were used: an *in-silico* dataset of simulated photoplethysmogram waves for healthy male participants (25–75 years old); an *in-vivo* dataset containing 10-min photoplethysmogram recordings from 57 healthy subjects at rest (18–39 or > 70 years old; 53% female); and an *in-vivo* dataset containing photoplethysmogram recordings collected for 4 weeks from a single subject, in daily life. The best-performing indices from the *in-silico* study (5/48 fiducial point analysis and 6/49 Symmetric Projection Attractor Reconstruction) were applied to the *in-vivo* datasets.

**Results:**

Key fiducial point analysis and Symmetric Projection Attractor Reconstruction indices, which showed the greatest differences between groups, were found to be consistent across datasets. These indices were related to systolic augmentation, diastolic peak positioning and prominence, and waveform variability. Both fiducial point analysis and Symmetric Projection Attractor Reconstruction techniques provided indices that supported the classification of age and physical activity, but not sex.

**Conclusions:**

Both fiducial point analysis and Symmetric Projection Attractor Reconstruction techniques demonstrated utility in identifying cardiovascular differences between individuals and within an individual over time. Future research should investigate the potential utility of these techniques for extracting information on fitness and disease, to support healthcare-decision making.

## Introduction

The photoplethysmogram (PPG) signal is primarily used for monitoring heart rate (HR) and blood oxygen saturation (SpO_2_)^1–4^ in both hospital and community-based patients. These indices are easily acquired from routinely available non-invasive clinical or commercial devices. Some smart wearables are capable of monitoring physiology during exercise^5,6^ and identifying atrial fibrillation.^7,8^ The recent surge in the popularity of commercially available PPG-based wearable devices demonstrates a demand for continuous, personal physiological monitoring, in both clinical and consumer settings. This may support earlier alerts to identify and expedite interventions for individuals at a higher risk of cardiovascular disease.

Here, we evaluated two different waveform analysis techniques: fiducial point analysis (FPA)^4,9,10^ and Symmetric Projection Attractor Reconstruction (SPAR)^11–15^ to exemplify the capacity of PPG waveform analysis, beyond that of standard extracted indices such as HR or SpO_2_. We hypothesised that the additional quantification of morphological and variability changes in the PPG, through FPA- and SPAR-generated indices, could provide further indicative information about cardiovascular health. These indices were investigated using one *in-silico* and two *in-vivo* non-patient datasets, for the classification of cohorts according to age, sex and physical activity.

Chronological aging is characterised by structural and functional changes within the vasculature, the rate of which varies from individual to individual.^16^ Accelerated vascular aging is a risk factor for cardiovascular disease^17^ and there is great interest in using the PPG signal to identify vascular aging biomarkers, to support earlier detection, in turn, expediting clinical management.

Investigation of sex-specific differences is also important to ensure personalised approaches for future healthcare technologies. Despite recent positive changes, most cardiovascular clinical studies have a male predominance, suggesting outcomes may not be generalisable to females. Sex was therefore considered an important biological variable for this study. Lastly, physical activity is known to impact cardiovascular performance, but separately, motion artefacts influence the quality of PPG recordings, particularly in community monitoring settings. We recognised the importance of testing the extremes of recorded signal PPG quality using both laboratory and community data within the present study to identify any challenges and method limitations.

The studies in this paper were therefore designed to demonstrate the sensitivity and applicability of the FPA and SPAR methods through the classification of age, sex and physical activity to be more than simple dataset classes, but rather important biological features that can support healthcare decision making.

### Fiducial point analysis

**Figure 1.**
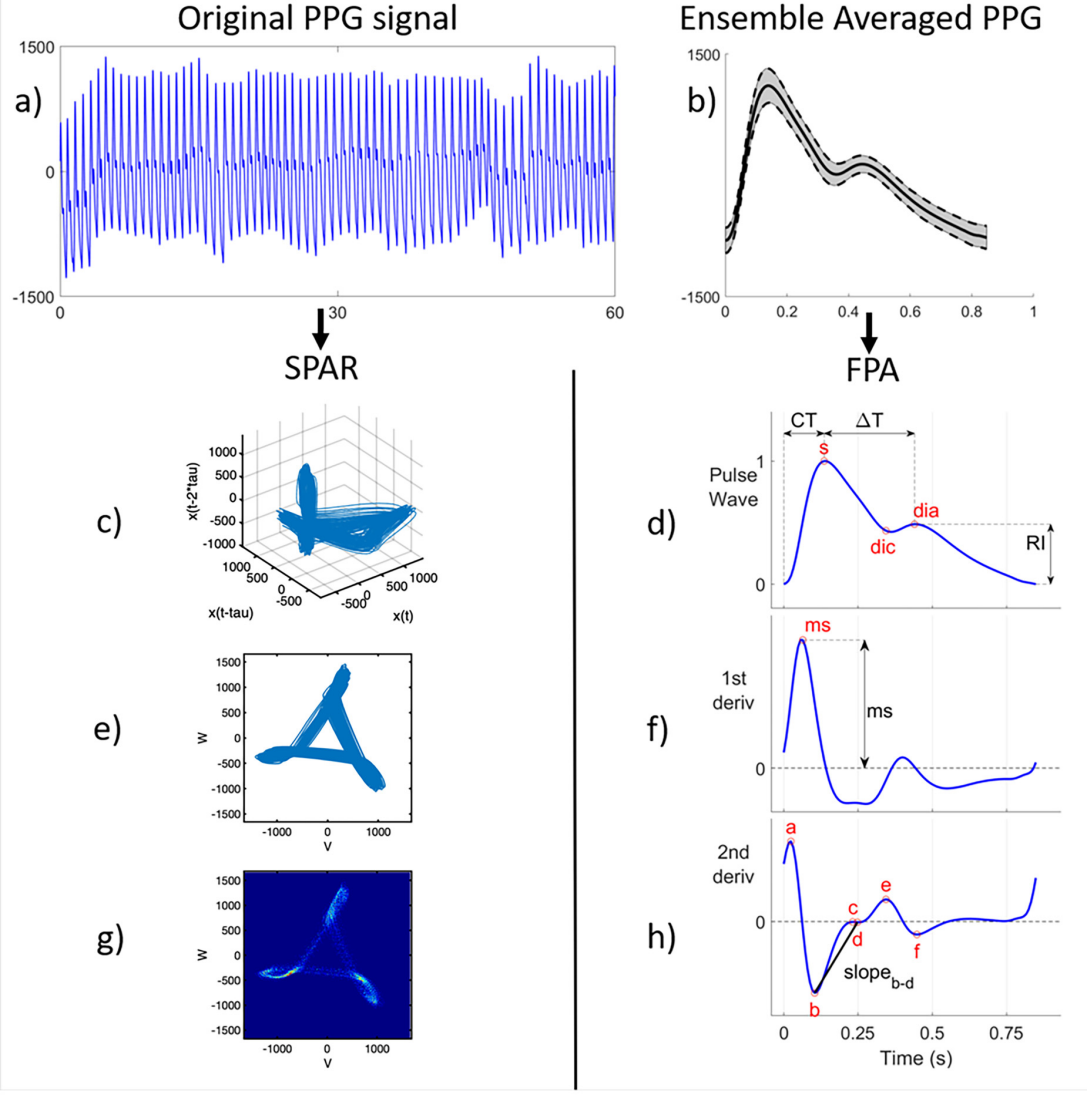
Methodology for analysing* in-vivo *PPG signals. PPG data segments (a) were pre-processed to detect individual beats and produce an ensemble-averaged PPG waveform (b). The original PPG data segment was analysed using the SPAR^11–13^ technique (left) and high-quality PPG pulses with the FPA^4,9,10^ technique (right). SPAR transforms a continuous PPG signal in time (a) to a 3D shape (c) projected down onto a 2D plane (e) from which a density map (g) highlights variability and from which indices can be calculated. FPA identifies fiducial points in PPG waveforms (b) and their first (f) and second (h) derivatives calculating indices from the timings and amplitudes of these points.

PPG: photoplethysmogram; VORTAL: vortal dataset; PPGD: PPG diary; SPAR: symmetric projection attractor reconstruction; FPA: fiducial point analysis; 3D: three-dimensional; 2D: two-dimensional.

FPA^4,9,10^ identifies specific points of interest on the pulse wave (or its derivatives) and calculates pulse wave indices from the timings and amplitudes of these points (Figure 1)*.* Larger segments of PPG recordings, captured over minutes or hours are split into windows (Figure 1(a)), individual beats are identified, and the ensemble is averaged for visualisation purposes (Figure 1(b)). Individual PPG pulse waves or the ensemble mean are then analysable by FPA (Figure 1(d), (f) and (h)). Points ‘*a*’ to ‘*f*’, (Figure 1(h)) are examples of fiducial points on the second derivative PPG. Pulse wave indices across the original PPG waveform and subsequent derivatives, have been found to provide clinically useful information about an individual's cardiovascular health.^4,9,10^ Two examples used in this study include the augmentation index (AI), calculated from the ratio of the early and late systolic peaks of the PPG waveform, and second derivative PPG indices calculated from the fiducial points ‘*a* to *f*’ which relate to arterial stiffness and vascular aging.^4,9,18–20^ In this study, a total of 48 FPA pulse wave indices were calculated for a comprehensive analysis of the PPG as previously described.^9,10^ Among these, five key indices were identified and used to perform more detailed analyses, with further information regarding their morphological interpretation found in Supplemental Table S1. The method is sensitive to electrical noise and motion artefacts in the input data; however, it can calculate indices from non-consecutive ‘high quality’ beats.

### Symmetric projection attractor reconstruction

The SPAR method was designed to calculate indices describing morphology and variability for any non-stationary, approximately cyclic signal (Figure 1(a)), by transforming windowed time-series data into a two-dimensional (2D) projection as previously described.^11–13^ Briefly, for each window of continuous PPG data, the average cycle length is calculated to determine a time delay, which is then used to select three equally spaced points in the waveform. The time delay auto-adjusts for each data window, such that it always corresponds to one-third of the average cycle length. The selected points are then shifted along the wave and their values are captured as numerical variables. These variables are then replotted as coordinates in three-dimensional (3D) phase space (Figure 1(c)). This 3D phase space is projected onto a 2D plane, which factors out baseline wander from the original signal (Figure 1(e)) and is termed an ‘attractor construct’ or ‘SPAR projection’. Each beat of the PPG signal generates one loop of the attractor about the origin of the 2D plane, which visually highlights subtle changes in the waveform‘s morphology. The attractor plot has an approximate three-fold rotational symmetry about the origin and is converted to a colour density map (Figure 1(g)).^11,12^

The shape of the attractor construct can be quantified in different ways, such as measuring the attractor central opening, arm width, symmetry, and attractor rotation angle and these indices correlate with specific features on the PPG waveform's morphology. The attractor's colour and density distribution provide information on variability, with zones of higher density (red) correlating with lower wave-to-wave variability. Some indices are more physiologically translatable than others. The SPAR method thus provides a qualitative at-a-glance image, from which quantitative indices can be calculated. The benefits of SPAR include its indifference to the input signal type (provided it is approximately cyclic), its use of the entire raw data (which minimises pre-processing bias introduction), its resistance to baseline wander (supporting application to real-world data), and its ability to amplify smaller changes in PPG morphology and variability, as larger visual changes on the attractor construct which can then be quantified. In this study, a total of 49 indices were calculated from the attractor constructs, of which six key indices were identified, for detailed analyses. Additional information regarding their interpretability in relation to the attractor morphology can be found in Supplemental Table S2.^11–13^

Unlike FPA, which can use discontinuous pulse waves, the SPAR index calculation in this article was performed with an input of ∼50–100 consecutive pulse waves. Therefore, it could be disrupted if frequent non-physiological artefacts, such as electrical noise or Bluetooth connection loss, corrupted the cyclic signal.^11^ Newer iterations of SPAR analysis could use non-consecutive segments of data but have not yet been implemented. Attractor quality indices could also be applied as a pre-processing step, to pre-select only cyclic data from the raw signal.

## Methods

This study is a retrospective analysis of one* in-silico* and two *in-vivo* human PPG datasets, with characteristics described in Table 1.

**Table 1.** Population characteristics of the three datasets used in this study: pulse wave database (PWDB^10^), clinical data subset of the vortal dataset (VORTAL^21^ and NCT01472133) and PPG diary (PPGD^22^).

Characteristic	PWDB	VORTAL	PPGD
Population size	3837	57	1
Age (years)	25, 35, 45, 55, 65, 75	18–39 >70	18–39
Number per cohort	712, 684, 654, 641, 588, 558	41 (21F, 20M) 16 (9F, 7M)	1
Sex	Male	53% female	Male
Data type	*In-silico*	*In-vivo*	*In-vivo*
Recording environment	Computer simulation	Clinical Laboratory	In-community
Signal type	Single cycle	Continuous	Continuous
Sampling frequency (Hz)	500	125	100

## Participants

### Pulse wave database (PWDB): In-silico data

The PWDB^10^ contains a population of thousands of virtual male subjects ranging from 25 to 75 years old, separated by a decade. For each subject, pulse wave signals were simulated in the larger arteries of the head, thorax, and limbs using one-dimensional blood flow modelling. Downstream vessels were represented by three-element Windkessel models, enabling the calculation of PPG signals in various body positions, including wrist, finger and ankle.^10^ As such, the* in-silico* PPG signals in PWDB were generated by solving the physical equations derived from the principles of conservation of mass and linear momentum. PWDB allows end users to identify subjects with different cardiac/vascular parameters (± 1 SD) compared to the baseline subjects, for each of the six age groups, to model their impacts on the waveform, within their corresponding age group. For example, changing the heart rate alters the cardiac output, which subsequently impacts the pulse wave propagation and reflection throughout the arterial network, resulting in different PPG wave morphologies. The model's parameters were based on literature values from healthy male subjects.^10^ This study used PPG signals from the left radial site, to approximate the location of a wrist-worn PPG device *in-vivo*. Further details of the PWDB dataset, which is publicly available (DOI: 10.5281/zenodo.3275625), along with methods and plausibility criteria used to simulate pulse waves, are published elsewhere.^10,23,24^

### VORTAL dataset: In-vivo data recorded in a laboratory

The VORTAL dataset (NCT01472133) is an *in-vivo* dataset collected in a clinical laboratory setting, containing data from 57 non-patient volunteers^21^ (Table 1). The subjects were split into cohorts based on age. The first two groups contained 21 female and 20 male subjects aged 18–39. The last two groups contained 9 female and 7 male subjects aged > 70 years old. Each subject had PPG signals recorded at the finger by a pulse oximeter whilst lying supine for ∼10 min.^21^

### PPG diary (PPGD): In-vivo data recorded in daily life

The PPGD dataset contains PPG data from one in-community male subject, recorded continuously, across a total of 4 weeks using the SmartCare wrist-worn pulse oximeter (SmartCare Analytics Ltd, London, UK).^22^ This dataset aims to replicate the type, quality, and quantity of data that might be collected from a typical user of a commercial smart wearable device. The dataset includes multiple labelled activities from which three were chosen: sleeping, seated computer work, and exercise [badminton]. These activities were specifically selected to reflect instances of common postures and physiological states in daily life. Notably, supine (sleeping – rest), seated (computer work) and standing active (badminton). The dataset contained gaps in recording, for reasons such as lost Bluetooth connection, device recharging and rest days when the device was not worn. This dataset is publicly available at (DOI: 10.5281/zenodo.3268500).^22^

A summary of each database's characteristics can be found in Table 1.

## Data processing and statistical analysis

FPA and SPAR analyses were performed on unfiltered data, using MATLAB version 2023a (MathWorks Ltd). FPA indices were computed using the PulseAnalyse^10^ toolbox and SPAR indices were computed with bespoke SPAR software. Statistical analyses were performed with bespoke software based on MATLAB version 2023a (MathWorks Ltd).

### In-silico PWDB

Percentage differences stemming from single *in-silico* cardiac/vascular parameter modifications and population-wide, age-dependent trends were calculated using all available FPA and SPAR indices. This first study identified the best-performing morphology indices which were subsequently applied to the *in-vivo* datasets.

### In-vivo VORTAL and PPGD

Key FPA and SPAR morphology indices identified from PWDB along with additional SPAR variability indices, were applied to VORTAL and PPGD. Data were analysed using univariate Receiver Operating Characteristic (ROC AUC) classification performance to compare across age and sex for VORTAL, and across activity (sleep, computer work and exercise) for PPGD. An arbitrary classification threshold of ROC AUC > 0.85 was considered to be biologically significant.

## Main outcome measures

### In-silico PWDB

A systematic alteration of individual cardiac and vascular parameters by ± 1 SD was carried out to model their impact on PPG pulse wave morphology and attractor constructs in the virtual 25-year-old baseline subject.^10^ The parameters were heart rate, stroke volume, left ventricular ejection time, vessel diameter, pulse wave velocity, and mean arterial pressure. The equivalent effects of aging were considered separately, using pulse waves from the 25-, 35-, 45-, 55-, 65- and 75-year-old cohorts, which were obtained by combining variations in both cardiac and vascular properties as observed in the clinical literature.^10^ Resultant PPG waveforms were extracted from the PWDB database and corresponding attractor constructs were generated. Changes to the PPG waveform and corresponding attractors were quantified in detail using the best-performing FPA and SPAR indices.

### In-vivo healthy human volunteer study (VORTAL)

The VORTAL^21^ dataset was split into four categories: ‘Young Female’, ‘Elderly Female’, ‘Young Male’ and ‘Elderly Male’. Representative 60-s PPG signals were manually selected for each individual and key FPA and SPAR indices were calculated and compared.

### In-vivo in-community male subject – PPGD

Three identified activities of PPGD,^22^ were analysed: ‘sleeping’, ‘computer’ and ‘exercise’ representing three common postures and physiological states in daily life. A secondary consideration was the recorded signal quality. ‘Sleeping’ in the supine position was defined as the baseline, due to a minimisation of motion artefacts, followed by ‘computer’ – seated and ‘exercise’ – standing. Similarly, despite significant signal artefacts and challenges to PPG analysis found in the ‘exercise’ data, we identified regions of the recording minimally affected by wrist motion artefact, but still captured the cardiovascular changes associated with exercise. For ‘sleeping’ and ‘computer’, a representative 60-second PPG data window was manually selected from each day where the activity occurred. On some days, no usable data was available. For ‘exercise’, four replicates were used from the same day, due to the unsuitable data for FPA analysis on other days. After selecting usable windows, 19 ‘sleeping’, 17 ‘computer’ and 4 ‘exercise’ representative PPG traces were analysed, and corresponding attractors were generated and quantified.

## Results

We have presented all qualitative results within the main manuscript and corresponding quantitative graphs and analyses pertaining to SPAR and FPA indices, can be found in the Supplemental Material.

### Effects of changing cardiac and vascular properties in a 25 y.o. virtual subject

#### In-silico PWDB

**Figure 2.**
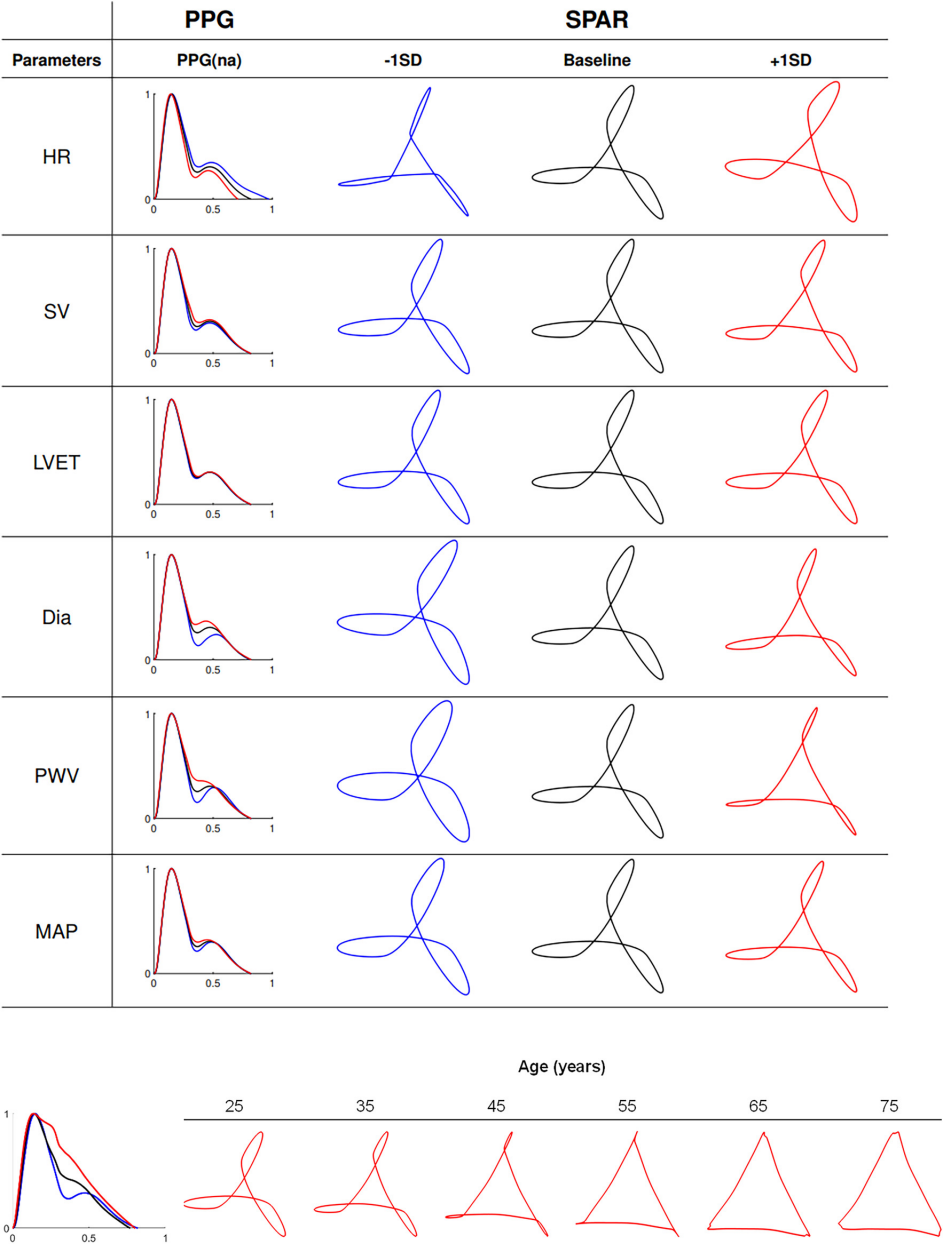
Upper panel: Effects of individual changes in cardiovascular parameters, on *in-silico* radial PPG wave morphology from PWDB.^10^ changes in PPG waveform morphology result from individual variation within one SD of the following parameters (−1SD in blue, + 1SD in red) around the baseline 25-year-old virtual subject (black lines) in PWDB: HR, SV, LVET, Dia, PWV, and MAP (second column). Corresponding attractor constructs are shown in the third to fifth columns. Lower panel: PPG waveform morphology of the 25-year-old (blue), 45-year-old (black) and 75-year-old (red) baseline subjects (left) and corresponding SPAR constructs for the baseline subjects of all age 6 groups (right).

PPG: photoplethysmogram; PWDB: pulse wave database; SD: standard deviation; HR: heart rate; SV: stroke volume; LVET: left ventricular ejection time; Dia: diameter; PWV: pulse wave velocity; MAP: mean arterial pressure.

Independent changes in both cardiac and vascular properties had a considerable effect on PPG wave morphology and subsequent attractor constructs (Figure 2, upper panel). Simulated variations in heart rate (HR), stroke volume (SV), vessel diameter (Dia), pulse wave velocity (PWV), or mean arterial pressure (MAP) mainly impacted PPG morphology around the diastolic peak (i.e. the peak appearing on the diastolic downstroke). Left ventricular ejection time (LVET) produced the fewest visually observable changes in PPG morphology. The magnitude of the diastolic peak increased with increasing SV, Dia, PWV, MAP and decreasing HR, with the greatest variations observed for HR, Dia and PWV. Additionally, increasing SV, Dia, PWV, and MAP, and shortening the beat period (by increasing HR) moved the diastolic peak closer to the systolic peak within the cardiac cycle.

Supplemental Table S3 shows the five FPA indices with the greatest percentage change variability from their baseline values*.* The second derivative PPG indices *c*/*a* and *d*/*a* exhibited the greatest percentage changes with individual property variation in SV and Dia. The same FPA indices changed to a lesser extent with HR, LVET and PVW.

SPAR constructs visually altered in morphology due to changes in PPG morphology (Figure 2, upper panel). There was an increased prominence of the incisura preceding the PPG's diastolic peak observed with decreasing Dia, PVW and MAP, and increasing HR, which was reflected as a widening of the edge looping effects at the corners of the triangular attractor construct and a change in the opening of the central core.

SPAR construct feature analysis identified five key indices with the greatest percentage changes, which quantified the ‘core opening’ (opening (5%)), ‘rotation’, ‘edge roundness’ (peak width), ‘outer arm looping (bandwidth) and rotational symmetry (symmetry). Morphological interpretation of SPAR features and percentage changes for each feature can be found in Supplemental Tables S2 and S3. Variability indices relating to SPAR were not considered in this example as, by definition, there is no variability present on a repeated single waveform.

SPAR indices changed with ±1SD Dia, consistent with FPA. Interestingly, SPAR showed larger changes with PWV and MAP, whilst FPA showed more pronounced changes to SV and Dia, as shown in Supplemental Table S3. These results highlight the complementarity of both FPA and SPAR methods, amplifying distinct changes, in response to different simulated cardiac and vascular property variations.

### Effects of aging on PPG wave morphology

#### In-silico PWDB

The diastolic peak on the PPG was attenuated with aging, resulting in reduced outer edge looping, evolving into a more open inner triangle in attractor constructs (Figure 2, lower panel). We demonstrate the FPA key indices *AI* and *d/a* changed in an age-dependent manner whilst *c/a* peaked and *e/a* troughed, at 55 years and *IPAD* showed a marginal age-dependent reduction between 35 and 65 years, as shown in Supplemental Figure S1.

For SPAR, the ‘opening’ increased whilst ‘bandwidth’ decreased, with age, whilst the remaining indices (rotation, peak width and symmetry) showed some age-dependent changes with inflections around 35–55 years of age, as shown in Supplemental Figure S1.

#### In-vivo healthy human volunteer study (VORTAL)

**Figure 3.**
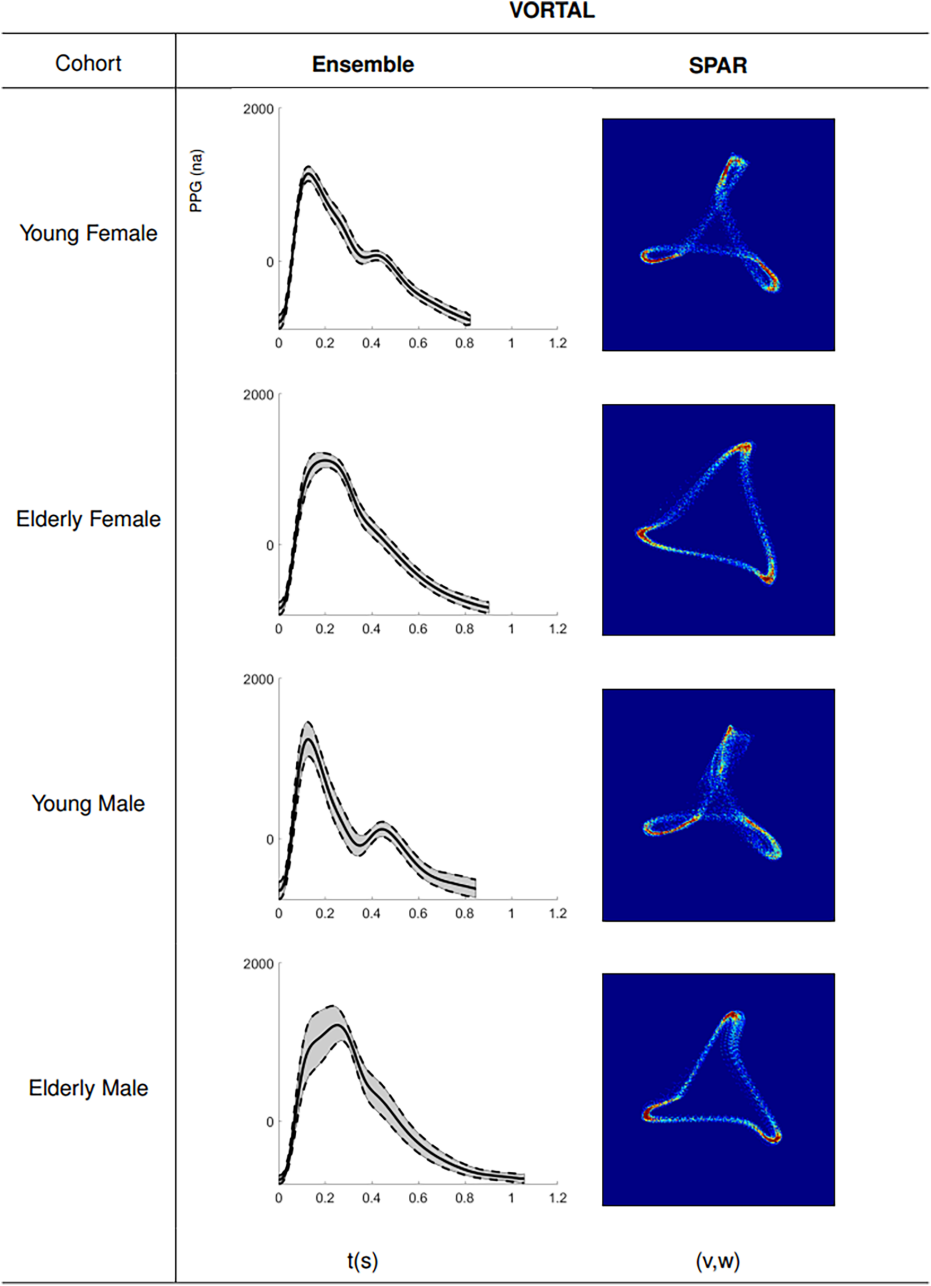
Exemplar PPG wave morphologies of different age and sex study groups from the *in-vivo *VORTAL^21^ dataset, analysed by ensemble averaged PPG and SPAR techniques. Each row corresponds to one of the four cohorts identified from VORTAL. Middle column: represents an ensemble-averaged 120 s segment of PPG data from a randomly selected subject within the cohort. Right column: represents the corresponding attractor construct.

PPG: photoplethysmogram; VORTAL: vortal dataset; SPAR: symmetric projection attractor reconstruction.

Vascular aging was subsequently studied* in-vivo* using the clinical dataset, VORTAL, with representative ensemble averages and attractors shown in Figure 3.

Quantitatively, the FPA method largely confirmed the observations from the* in-silico* PWDB, whereby *c*/*a* and *d/a* showed high classification accuracy between young and elderly cohorts (ROC AUC > 0.95) with *AI* and *IPAD* performance classification marginally poorer (ROC AUC 0.85–0.9), shown by the left column boxplot analyses in Supplemental Figure S2.

For SPAR analysis, attractor constructs of younger subjects showed an outer edge looping which receded, to create a more open and less variable triangle, in the elderly subjects, consistent with PWDB *in-silico* observations. This age-dependent change correlated with the attenuation of the diastolic peak on the PPG downstroke (Figure 3). Consistent with PWDB findings, SPAR ‘opening’ and ‘bandwidth’ showed the highest classification accuracy with aging (ROC AUC > 0.95). Other SPAR indices (‘rotation’ and ‘arm density’) also showed age-dependent changes but with marginally poorer classification accuracy (ROC AUC 0.85–0.95), as shown in the right column boxplots of Supplemental Figure 2.

We observed some changes between male and female subjects (notably in the IPAD and ‘rotation’ indices) (ROC AUC 0.9–0.95). The remaining Supplemental Figures S4 to S11 illustrate ensembled average waveforms and attractors from the individual 57 VORTAL participants, separated into cohorts according to sex, the split number indicates a larger cohort separated across multiple figures.

ROC AUC results for all studied indices in this database are shown in the leftmost section of Supplemental Table S4.

### Effects of physical activity on PPG wave morphology

**Figure 4.**
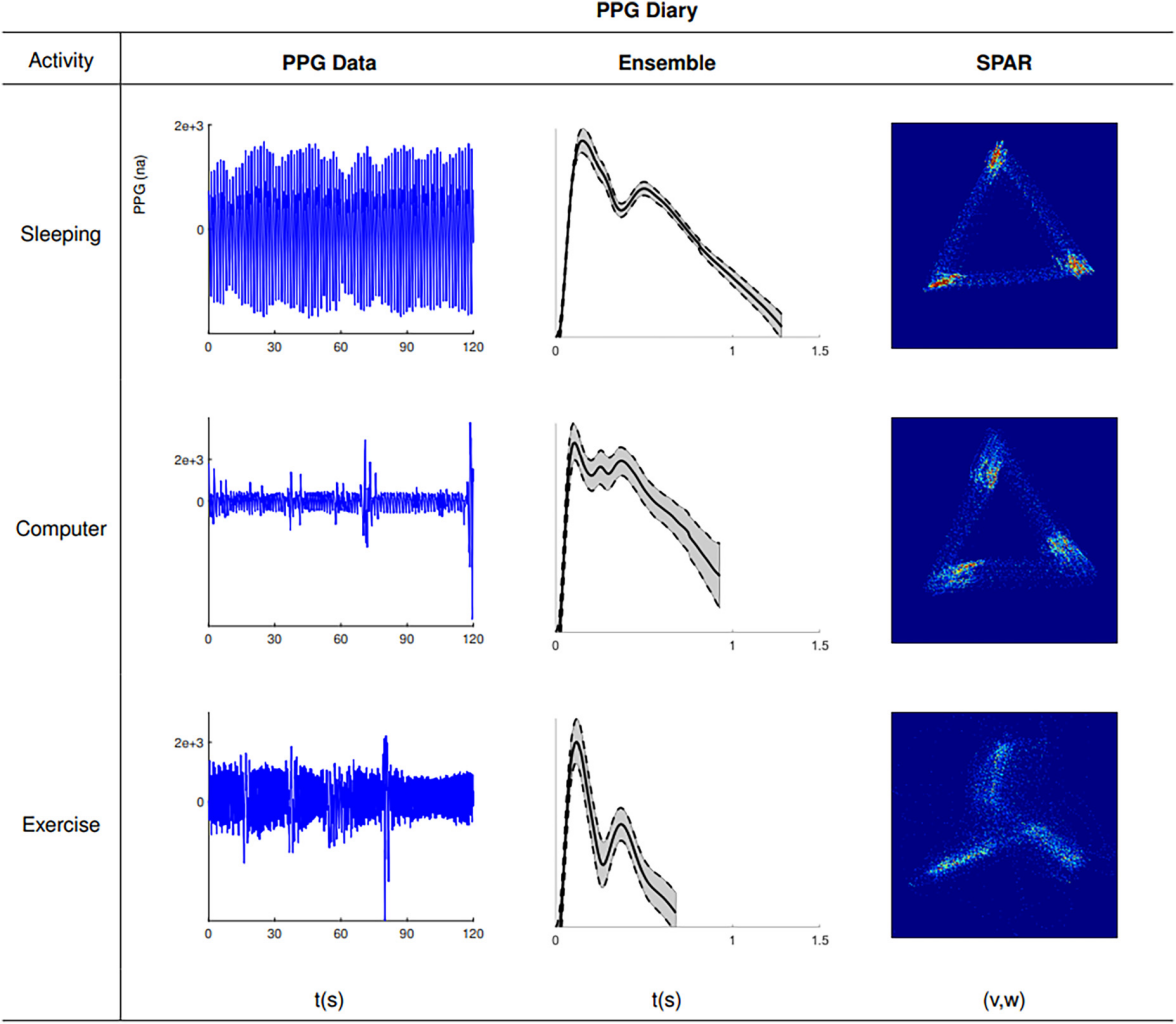
PPG data during the three selected activities from PPGD,^22^ was analysed by ensemble-averaged PPG and SPAR^11–13^ techniques. Left column: 120 s segments of PPG data are shown, middle column: ensemble averaged PPG waveforms derived from the PPG data input and the right column: corresponding attractor constructs.

PPG: photoplethysmogram; PPGD: PPG diary; SPAR: symmetric projection attractor reconstruction.

There was an observed increase in signal recording artefacts with the selected physical activities of the *in-vivo* in-community male subject – PPGD as compared to the recordings from VORTAL. Representative traces, ensemble average and attractors for sleeping, computer (low activity and seated posture) and exercise (highest activity and standing dynamic posture), from good quality data, are shown in Figure 4.

The FPA indices *IPAD*, *c*/*a* and *d*/*a* showed the highest classification performances between activities (ROC AUC 0.85-0.95) shown in the left column boxplots of Supplemental Figure S3, whereby *IPAD* decreased and *c/a* increased, with activity level, AI once again performed marginally poorer (ROC AUC 0.85–0.90).

SPAR successfully generated attractor constructs for each activity (Figure 4) and most notably, indices quantifying inner opening (‘opening’ and ‘bandwidth’) showed the highest classification performance (ROC AUC > 0.95) which correlated with an increased diastolic peak height on the PPG downstroke and an increased overall waveform variability with increasing levels of activity. SPAR analysis revealed significant activity-dependent changes (ROC AUC > 0.90) between sleeping versus seated computer work, seated computer work versus exercise, and sleeping versus exercise. These results are all illustrated in the right column boxplots of Supplemental Figure S3.

ROC AUC results for all studied indices in this database are shown in the rightmost section of Supplemental Table S4.

## Discussion

This study has demonstrated the complementary value of two pulse wave analysis methods (FPA and SPAR) which appraise PPG signals in distinct ways. FPA utilises high-quality beats within a window of data and can be applied to discontinuous data. In contrast, in this study, SPAR used continuous cyclic data to generate a quantifiable attractor construct. Despite these differences, they demonstrated the capacity for a comprehensive, sensitive and reproducible analysis of PPG waveforms, via consistently identified indices, across varied datasets. In this study, we demonstrated sensitive quantification and classification of cardiovascular change from PPG signals with age and physical activity, but not sex, across *in-silico* and *in-vivo* laboratory and *in-vivo* community environments, in non-patient subjects.

Specific advantages of both methods include their ability to analyse single pulse waves or longer windows of data, allowing end users to optimise the granularity of analysis, depending on the context.

Separately, SPAR generates an image representation of the raw time series data, visually amplifying morphological and variability changes at-a-glance. This may aid in the more sensitive detection of physiological change in time-critical clinical settings, such as intensive or emergency care or in community based higher risk patients, where detailed waveform analysis may not be feasible, or where patients may look otherwise unremarkable.

There are also method limitations. FPA relies on data of sufficiently high quality for accurate detection of fiducial points to be possible, and this constraint was highlighted by the limited usable data from PPG Diary – exercise data. For SPAR analysis, this motion artefact was less of an issue. Whilst we demonstrated clear differences between groups with certain SPAR indices, more specific indices relating to observed morphological features of the attractor construct (such as the outer arm looping), could be developed, which could more sensitively and specifically characterise the PPG waveform changes.

For aging, the FPA measures *AI*, *IPAD* and second derivative indices *c/a* and *d/a,* were identified* in-silico* as the best classifiers, consistent with arterial stiffening and increased luminal diameter associated with increased age, as described in the clinical literature.^4,9,10,18,19^ *In-silico* modelled vascular aging, resulted in the PPG diastolic peak moving closer to the systolic peak within the cardiac cycle. The age-related morphology changes observed *in-silico* were largely mirrored in the *in-vivo* VORTAL dataset, with second derivative measures showing statistically significant changes, confirming the clinical translatability of the PWDB *in-silico* database and consistent with published observations.^20,25^ With SPAR analysis, the effects of simulated aging from the *in-silico* PWBD were also mirrored in the *in-vivo* VORTAL dataset, whereby increased aging was associated with more open, less looped attractors, arising from attenuation of the diastolic peak on the downstroke.

The trajectory changes in FPA and SPAR indices noted from 40 years old, within the *in-silico* PWDB and *in-vivo* VORTAL datasets, are consistent with published observations^26^ and, by extension, the recognition that a focus on modifiable cardiovascular risk factors should be considered in people, before the age of 40.^27,28^

SPAR indices relating to waveform variability also showed age-dependent changes, whereby attractor constructs of elderly volunteers from the VORTAL dataset, showed lower waveform variability, as captured by higher density and narrower redder arms, compared to younger volunteers. These findings are consistent with well-established decreased heart rate variability observed with aging.^29^

The attenuation of the outer looping of attractor constructs with aging relates to the movement of the diastolic peak. This appears prominently on the diastolic downstroke in younger individuals but is likely being reflected earlier in the cardiac cycle in older individuals, where it is absorbed within the systolic peak, resulting in an increased systolic pressure. Whilst this phenomenon is already known and quantified using the established metric ‘Augmentation Index’ (*AI*) which measures the distance between the anacrotic notch and systolic peak*,* the attractor constructs amplify these waveform changes. As SPAR is resistant to baseline wander, it may overcome current challenges associated with the accurate detection of specific waveform points, such as the anacrotic notch, from real-world non-stationary clinical data. Indeed, in this study, AI was less accurate at classifying by age in the VORTAL dataset.

FPA and SPAR also successfully classified the physical activity changes in the* in vivo* PPG Diary dataset from a healthy individual. Once again, the prominence and position of the diastolic peak showed the greatest changes when transitioning from low-activity sleeping, to high-activity exercise. SPAR constructs amplified these changes, observed as a progressive closing of the attractors as activities intensified. As anticipated, higher physical activity resulted in higher waveform variability, visualised and quantified as a more diffuse attractor construct. This is consistent with previous studies investigating increased exercise loading on radial pulse waveforms^30^ but here, we present new ways to visualise and quantify these changes.

Sex differences in the PPG were less clear with both FPA and SPAR, and possible reasons are discussed below.

This study was subject to limitations. Firstly, the *in-vivo* VORTAL data set was underpowered for the FPA analysis considering sex, a consequence of relatively small sample sizes for both male and female participants, particularly when separated by age. Secondly, we had limited pre-existing computed indices for SPAR. Whilst the outer looping of the attractor provided striking visual differences with aging, this metric was not possible to accurately compute in this study, and this visual feature was not directly quantified. Instead, the relative ‘openness’ of the attractor was measured as a surrogate. Thirdly, the inclusion of physical activity as a key indicator demonstrated the current limitations in signal recording quality when measuring states such as exercise. In the future, the application of deep learning, both to signal processing of the PPG waveform and to the attractor construct image itself, may overcome some of these limitations.^31^

Overall, expanding the physiological information garnered from rudimentary PPG signals can support continuous tracking of the cardiovascular system, where alternate pulse wave monitoring via intra-arterial catheters, repeated cuff-based monitoring or arterial tonometry, is not feasible. The extraction of additional data from PPG wearables, could support earlier detection of physiological change, cardiovascular disease or track improvements after an intervention, for individuals in lower dependency and community settings. We propose the PPG signal can, and should, be used to extract more than heart rate and SpO_2_. In this proof-of-concept study, we demonstrate how the addition of SPAR and FPA software, could support a fuller use of the signal. For example, our results suggest SPAR and FPA's quantification of vascular aging, a phenomenon underpinned by arterial stiffness,^16^ could identify, and expedite primary prevention strategies in younger, at-risk individuals.

This potential use of a simple PPG signal, rather than the more complex instrumentation required for the widely accepted pulse wave velocity measurement, could support patient triage within primary care settings, overcoming time, cost and device limitations that have been reported.^32^ In conclusion, whilst both FPA and SPAR indices should be validated in larger datasets, our results demonstrated the potential application of FPA and SPAR analysis both clinical and in-community environments.

## Supplemental Material

sj-docx-1-cvd-10.1177_20480040231225384 - Supplemental material for Advanced waveform analysis of the photoplethysmogram signal using complementary signal processing techniques for the extraction of biomarkers of cardiovascular function Click here for additional data file. Supplemental material, sj-docx-1-cvd-10.1177_20480040231225384 for Advanced waveform analysis of the photoplethysmogram signal using complementary signal processing techniques for the extraction of biomarkers of cardiovascular function by Aristide Jun Wen Mathieu, Miquel Serna Pascual, Peter H Charlton, Maria Volovaya, Jenny Venton, Philip J Aston, Manasi Nandi and Jordi Alastruey in JRSM Cardiovascular Disease

sj-docx-2-cvd-10.1177_20480040231225384 - Supplemental material for Advanced waveform analysis of the photoplethysmogram signal using complementary signal processing techniques for the extraction of biomarkers of cardiovascular function Click here for additional data file. Supplemental material, sj-docx-2-cvd-10.1177_20480040231225384 for Advanced waveform analysis of the photoplethysmogram signal using complementary signal processing techniques for the extraction of biomarkers of cardiovascular function by Aristide Jun Wen Mathieu, Miquel Serna Pascual, Peter H Charlton, Maria Volovaya, Jenny Venton, Philip J Aston, Manasi Nandi and Jordi Alastruey in JRSM Cardiovascular Disease

sj-docx-3-cvd-10.1177_20480040231225384 - Supplemental material for Advanced waveform analysis of the photoplethysmogram signal using complementary signal processing techniques for the extraction of biomarkers of cardiovascular function Click here for additional data file. Supplemental material, sj-docx-3-cvd-10.1177_20480040231225384 for Advanced waveform analysis of the photoplethysmogram signal using complementary signal processing techniques for the extraction of biomarkers of cardiovascular function by Aristide Jun Wen Mathieu, Miquel Serna Pascual, Peter H Charlton, Maria Volovaya, Jenny Venton, Philip J Aston, Manasi Nandi and Jordi Alastruey in JRSM Cardiovascular Disease

sj-docx-4-cvd-10.1177_20480040231225384 - Supplemental material for Advanced waveform analysis of the photoplethysmogram signal using complementary signal processing techniques for the extraction of biomarkers of cardiovascular function Click here for additional data file. Supplemental material, sj-docx-4-cvd-10.1177_20480040231225384 for Advanced waveform analysis of the photoplethysmogram signal using complementary signal processing techniques for the extraction of biomarkers of cardiovascular function by Aristide Jun Wen Mathieu, Miquel Serna Pascual, Peter H Charlton, Maria Volovaya, Jenny Venton, Philip J Aston, Manasi Nandi and Jordi Alastruey in JRSM Cardiovascular Disease

sj-docx-5-cvd-10.1177_20480040231225384 - Supplemental material for Advanced waveform analysis of the photoplethysmogram signal using complementary signal processing techniques for the extraction of biomarkers of cardiovascular function Click here for additional data file. Supplemental material, sj-docx-5-cvd-10.1177_20480040231225384 for Advanced waveform analysis of the photoplethysmogram signal using complementary signal processing techniques for the extraction of biomarkers of cardiovascular function by Aristide Jun Wen Mathieu, Miquel Serna Pascual, Peter H Charlton, Maria Volovaya, Jenny Venton, Philip J Aston, Manasi Nandi and Jordi Alastruey in JRSM Cardiovascular Disease
