# Supplementary material for: Advanced waveform analysis of the photoplethysmogram signal using complementary signal processing techniques for the extraction of biomarkers of cardiovascular function
Source: JRSM Cardiovasc Dis. 2024 Feb 1;13:20480040231225384. doi: 10.1177/20480040231225384 (PMC10838030; doi:10.1177/20480040231225384)
Supplement: sj-docx-1-cvd-10.1177_20480040231225384 - Supplemental material for Advanced waveform analysis of the photoplethysmogram signal using complementary signal processing techniques for the extraction of biomarkers of cardiovascular function [file sj-docx-1-cvd-10.1177_20480040231225384.docx]

**Supplementary Figures**

| **FPA** |  | **SPAR** |
| --- | --- | --- |
|  |  |  |
| 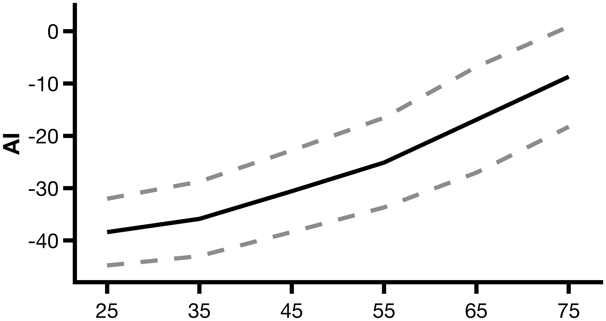 |  | 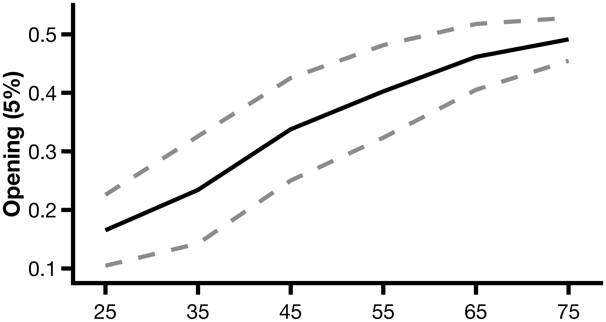 |
| 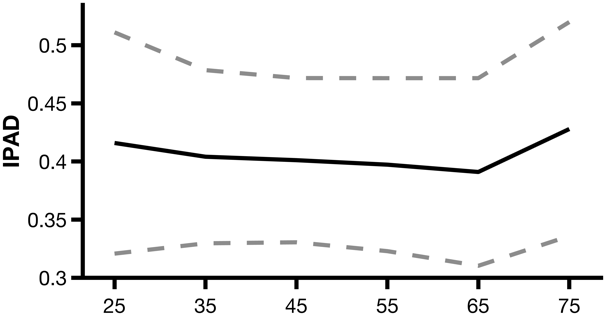 |  | 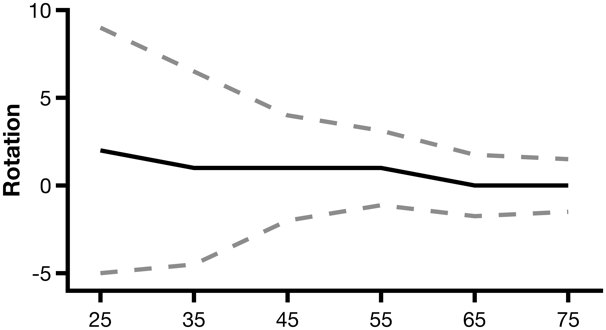 |
| 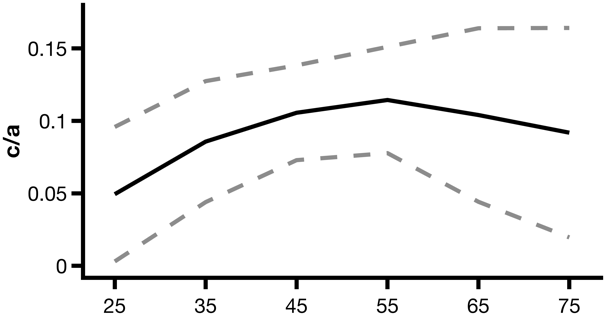 |  | 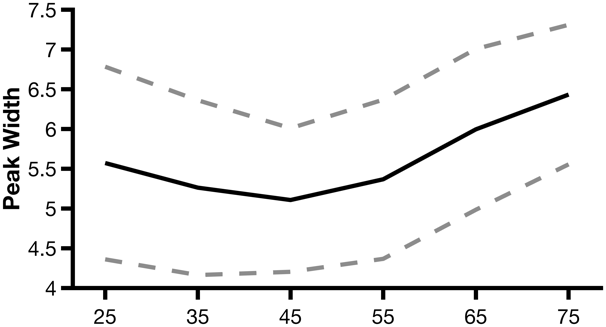 |
| 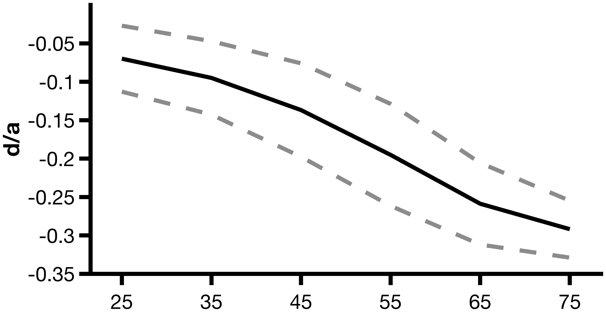 |  | 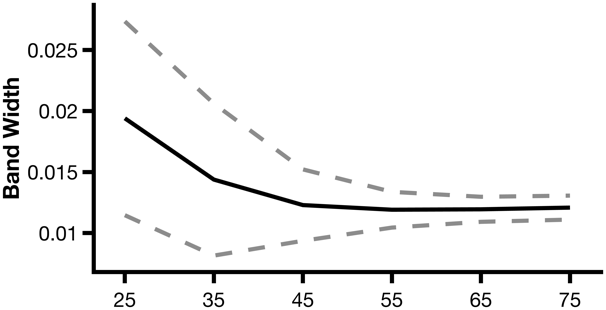 |
| 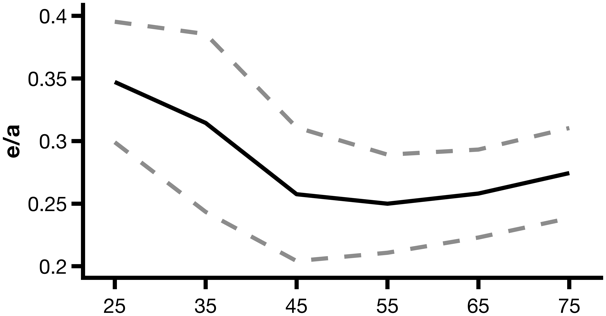 |  | 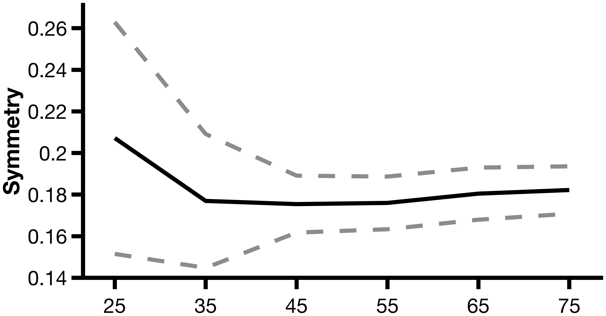 |
| Age (y.o.) |  | Age (y.o.) |
| **Figure S1:** Age-dependent changes of selected FPA^4,9,10^ (left column) and SPAR^11-13^ (right column) indices from all *in-silico* subjects of the PWDB database^10^. | | |

| **FPA** |  | **SPAR** |
| --- | --- | --- |
|  |  |  |
| 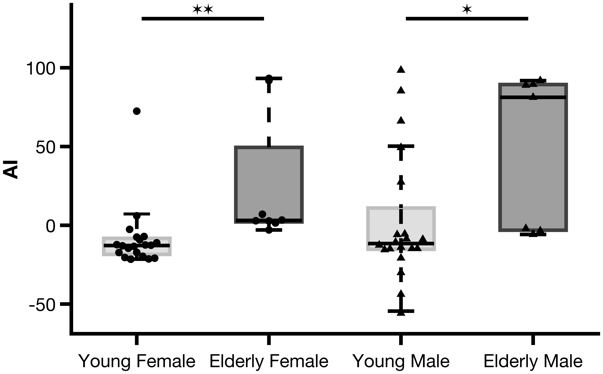 |  | 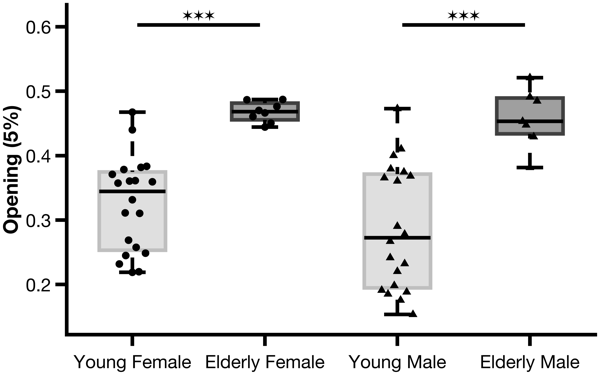 |
| 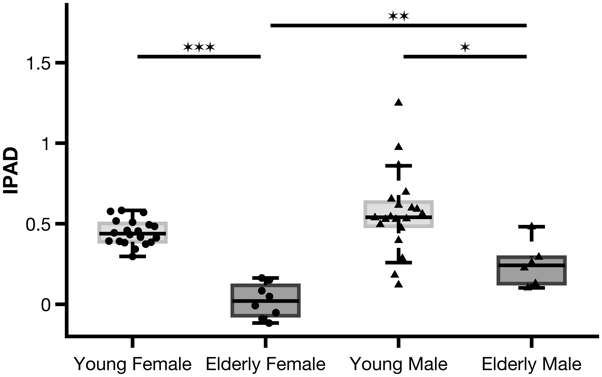 |  | 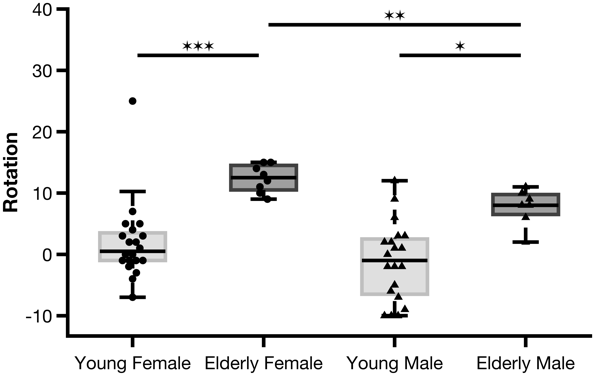 |
| 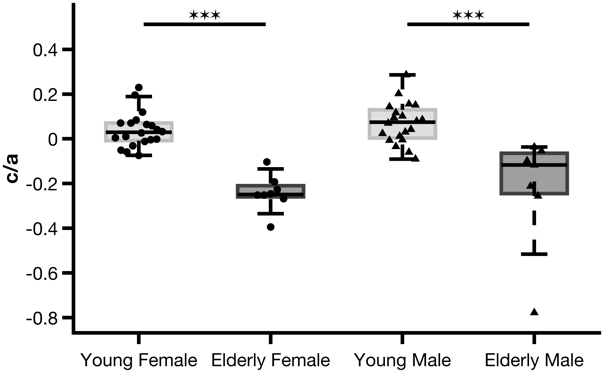 |  | 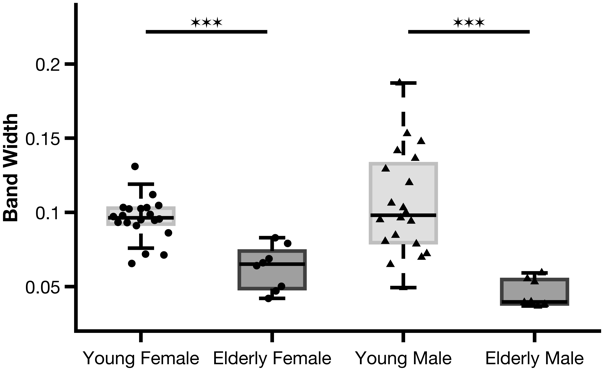 |
| 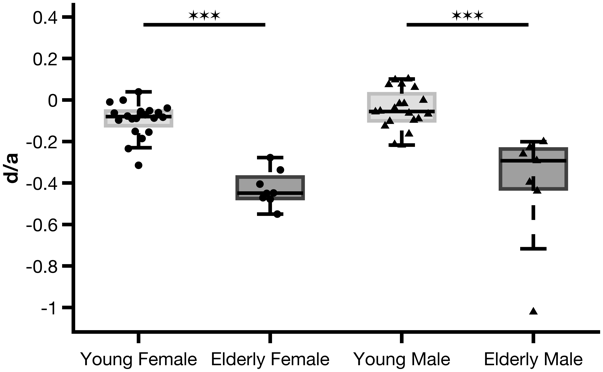 |  | 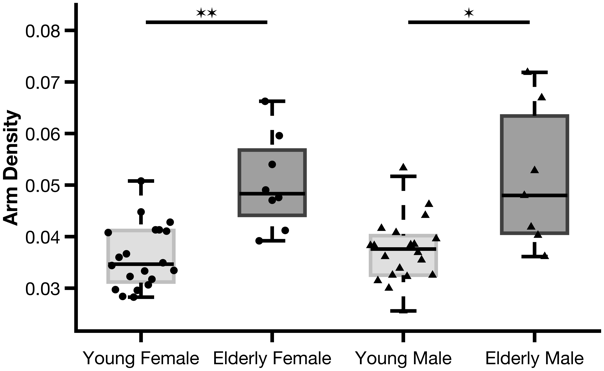 |
| **Figure S2:** Boxplot comparisons of best performing FPA^4,9,10^ (left column) and SPAR^11-13^ (right column) indices from 60-second data windows of the different VORTAL^23^ groups (x axes). Classification performances of all indices shown in Table S4. Markers above plots indicate results of a univariate logistic regression ROC AUC performance after internal validation. ✶✶✶ = ROCAUC > 0.95, ✶✶ = ROCAUC > 0.90, ✶ = ROCAUC > 0.85. Comparisons between groups with opposite sex and ages not shown. | | |
| **FPA** |  | **SPAR** |
|  |  |  |
| 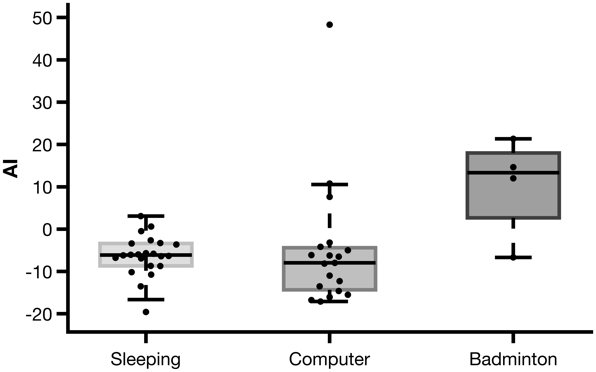 |  | 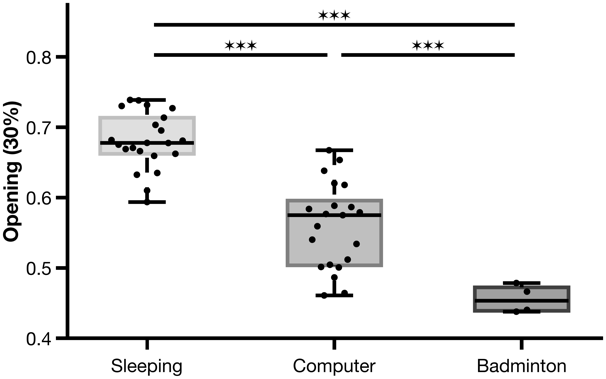 |
| 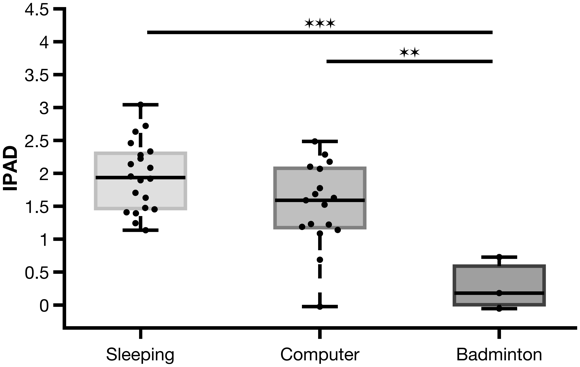 |  | 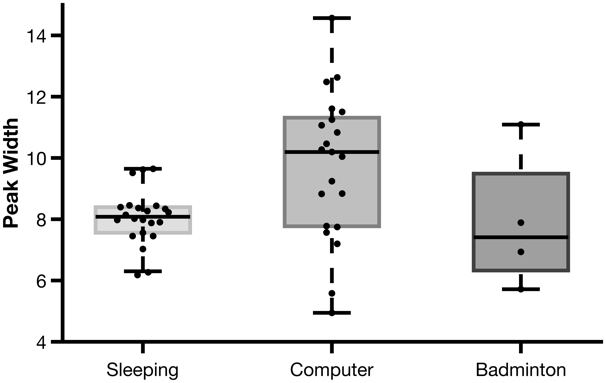 |
| 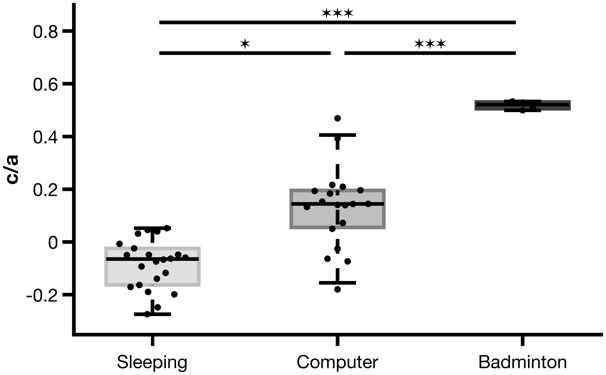 |  | 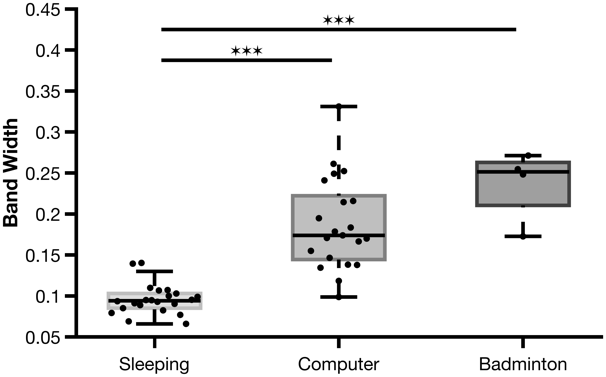 |
| 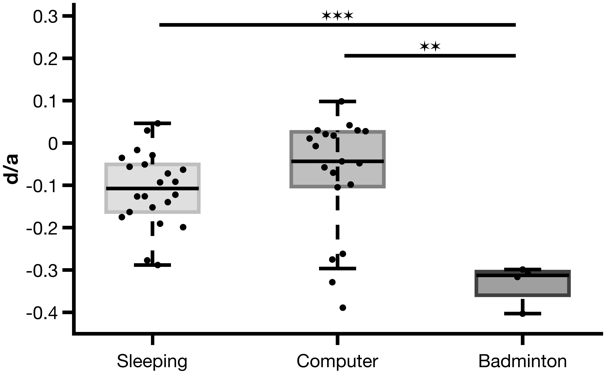 |  | 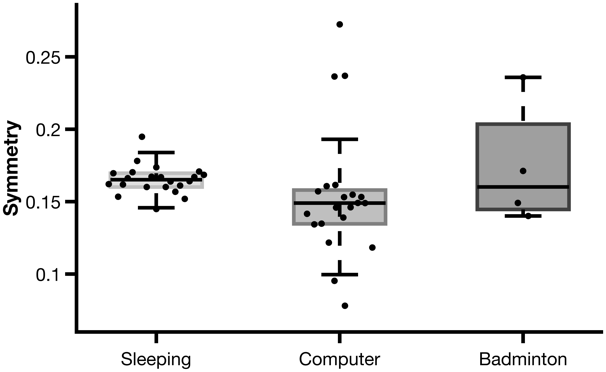 |
| **Figure S3:** Boxplot comparisons of best performing FPA^4,9,10^ and SPAR^11-13^ indices from 60-second data windows of the different PPG Diary (PPGD^24^) activities (x axes). Classification performances of all indices shown in Table S4. Markers above plots indicate results of a univariate logistic regression ROC AUC performance after internal validation. ✶✶✶ = ROCAUC > 0.95, ✶✶ = ROCAUC > 0.90, ✶ = ROCAUC > 0.85. | | |

**
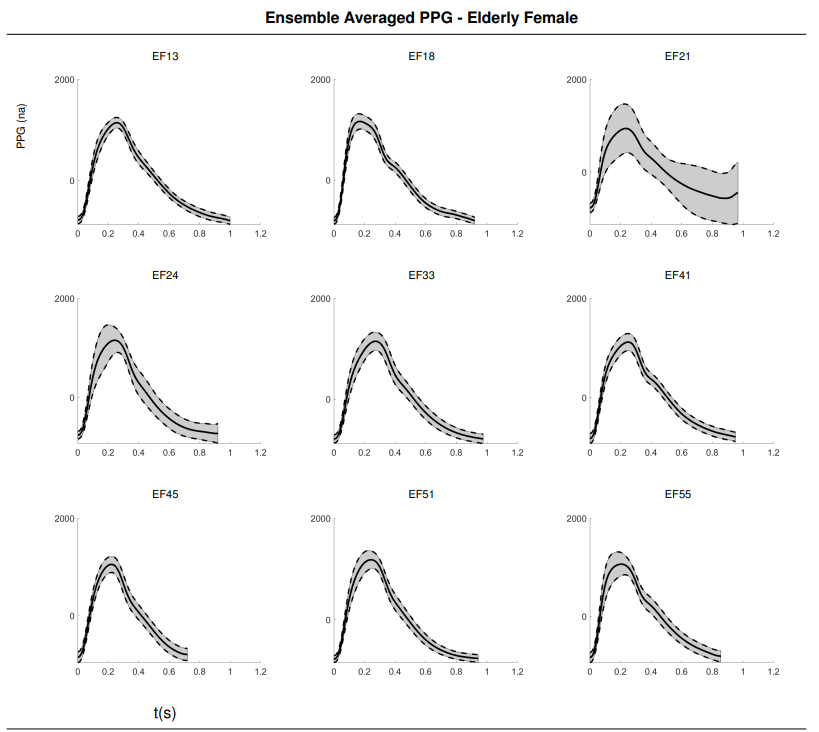
**

**Figure S4:** Ensemble average of 120-second segments from PPG signals of all elderly female subjects in VORTAL ^23^.

**
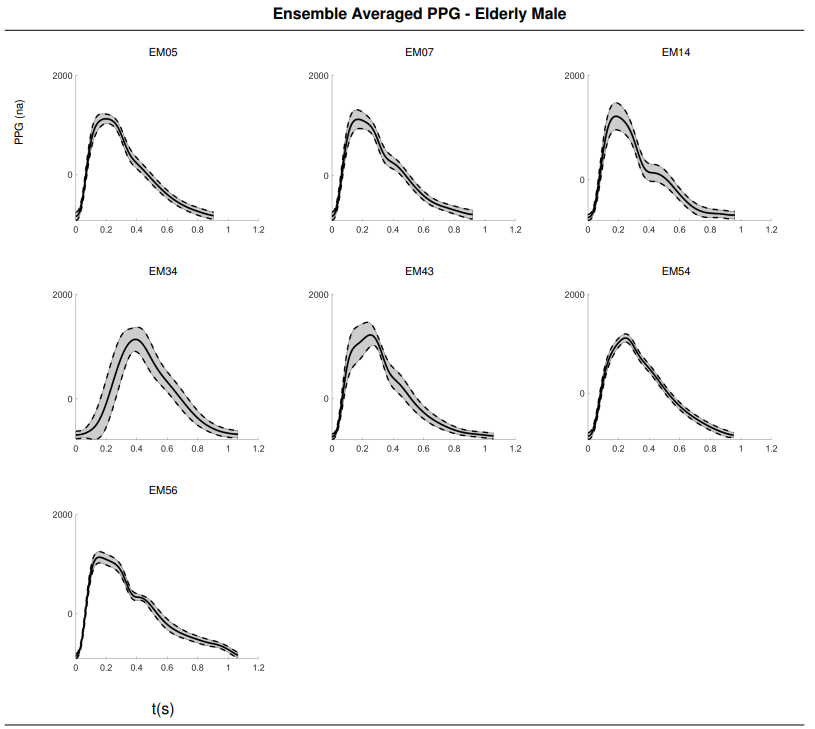
**

**Figure S5:** Ensemble average of 120-second segments from PPG signals of all elderly male subjects in VORTAL^23^.

**
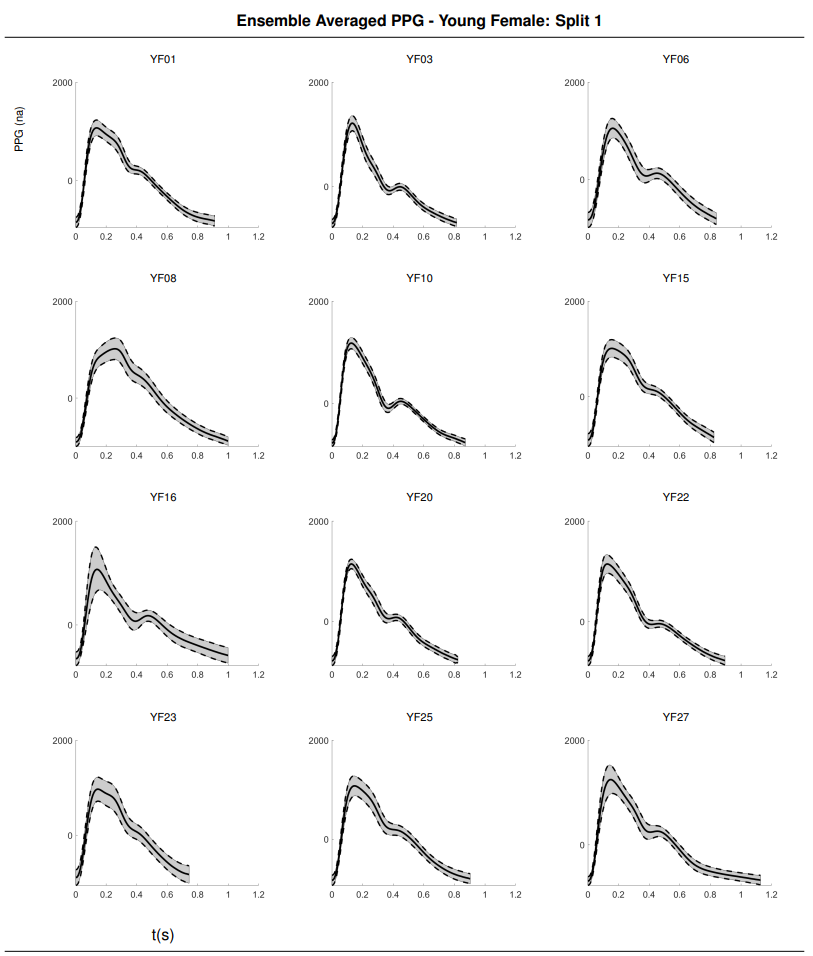
**

**Figure S6i:** Ensemble average of 120-second segments from PPG signals from the first 12 young female subjects in VORTAL^23^.

**
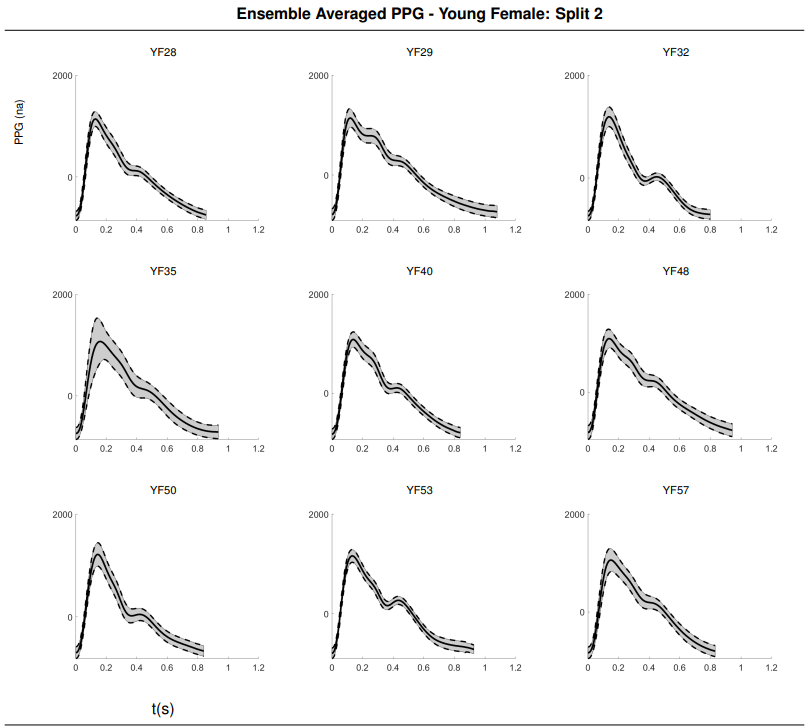
**

**Figure S6ii:** Ensemble average of 120-second segments from PPG signals, taken from last 9 young female subjects in VORTAL^23^.

**
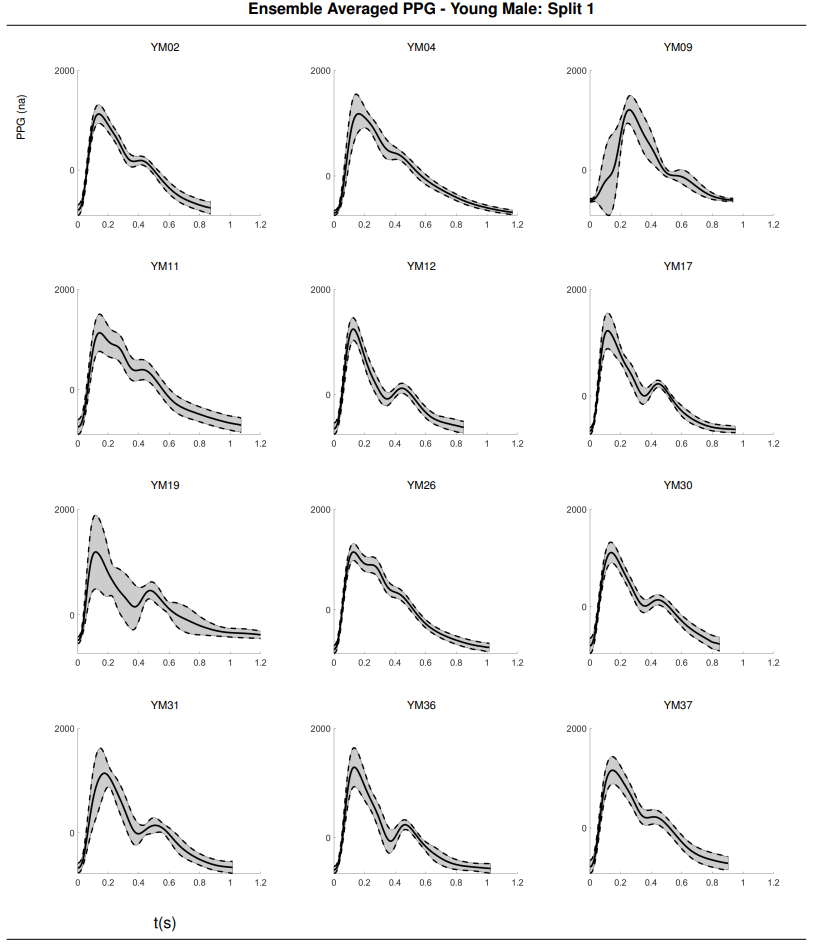
**

**Figure S7i:** Ensemble average of 120-second segments from PPG signals of first 12 young male subjects in VORTAL^23^.

**
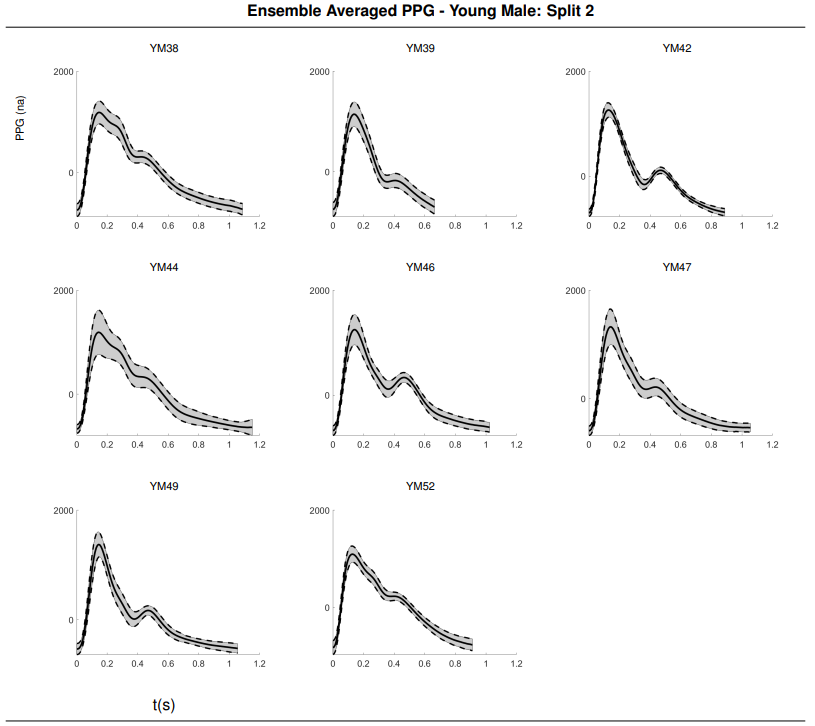
**

**Figure S7ii:** Ensemble average of 120-second segments from PPG signals of last 8 young male subjects of VORTAL^23^.

**
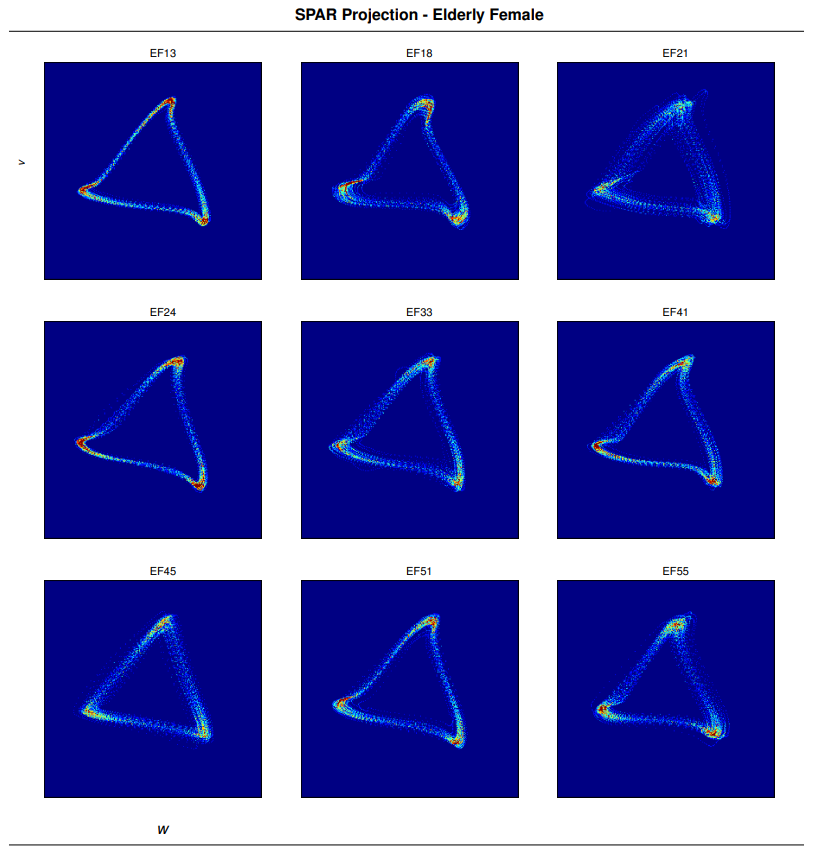
**

**Figure S8:** Attractor constructs of original input PPG data, taken from each elderly female subject from VORTAL^23^. Projections were created at 120-second window intervals and a random window per subject was selected for the figure.

**
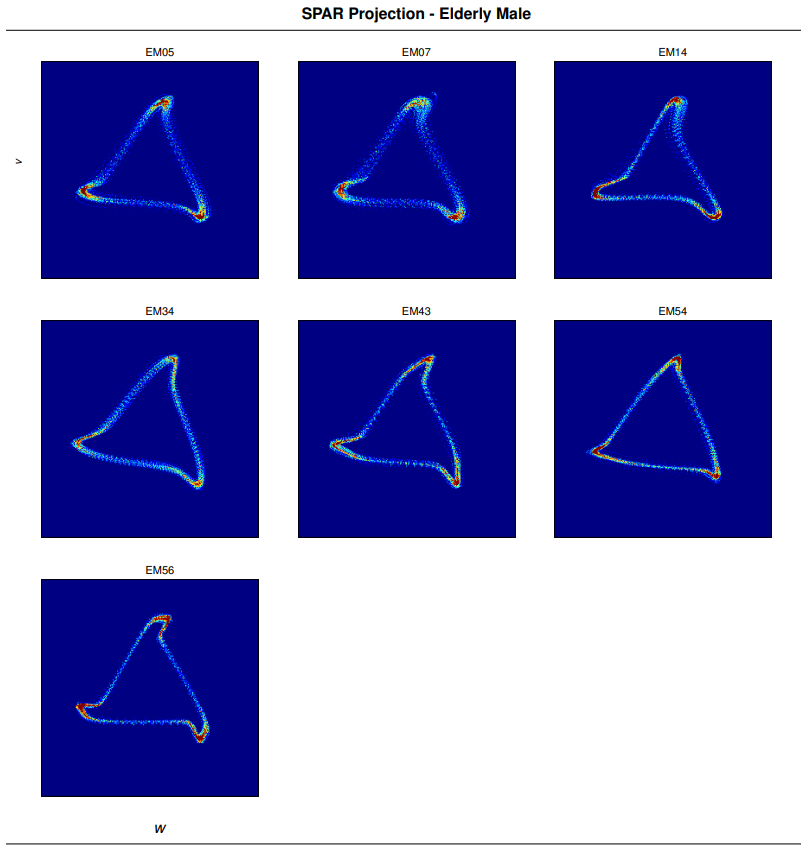
**

**Figure S9:** Attractor constructs of original input PPG data, taken from each elderly male subject from VORTAL^23^. Projections were created at 120-second window intervals and a random window per subject was selected for the figure.

**
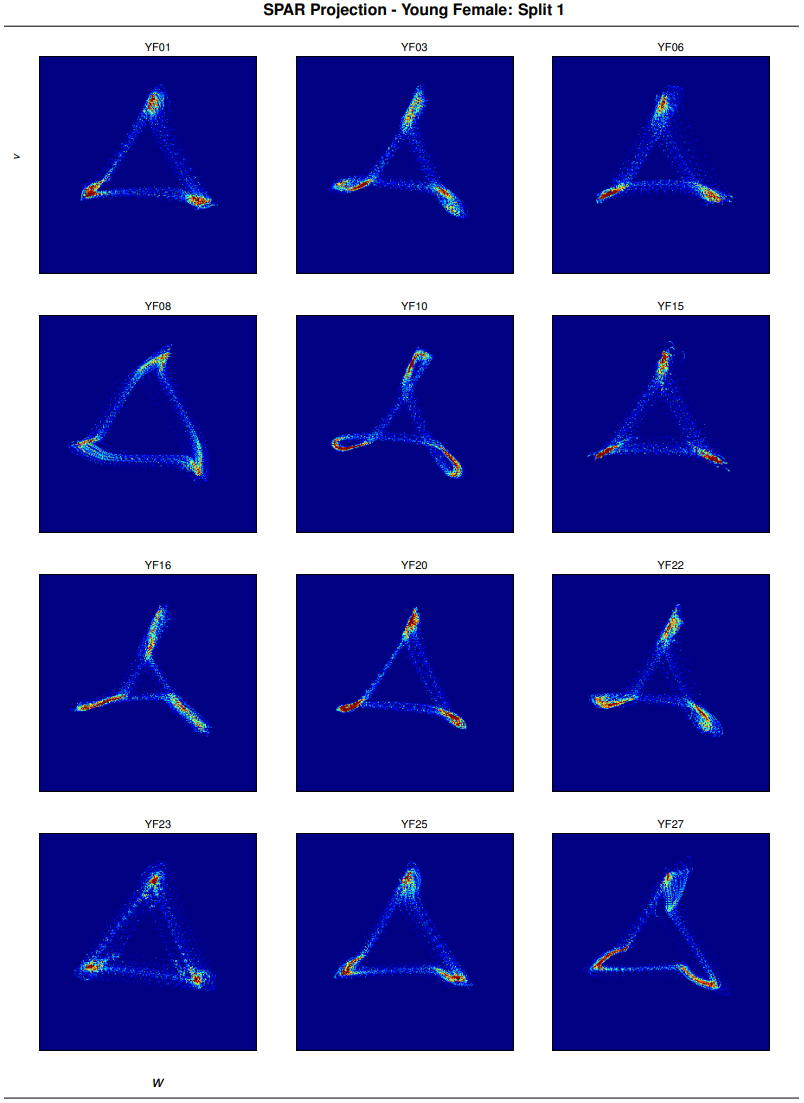
**

**Figure S10 i:** Attractor constructs of original input PPG data, taken from first 12 young female subjects of VORTAL^23^. Projections were created at 120-second window intervals and a random window per subject was selected for the figure.

**
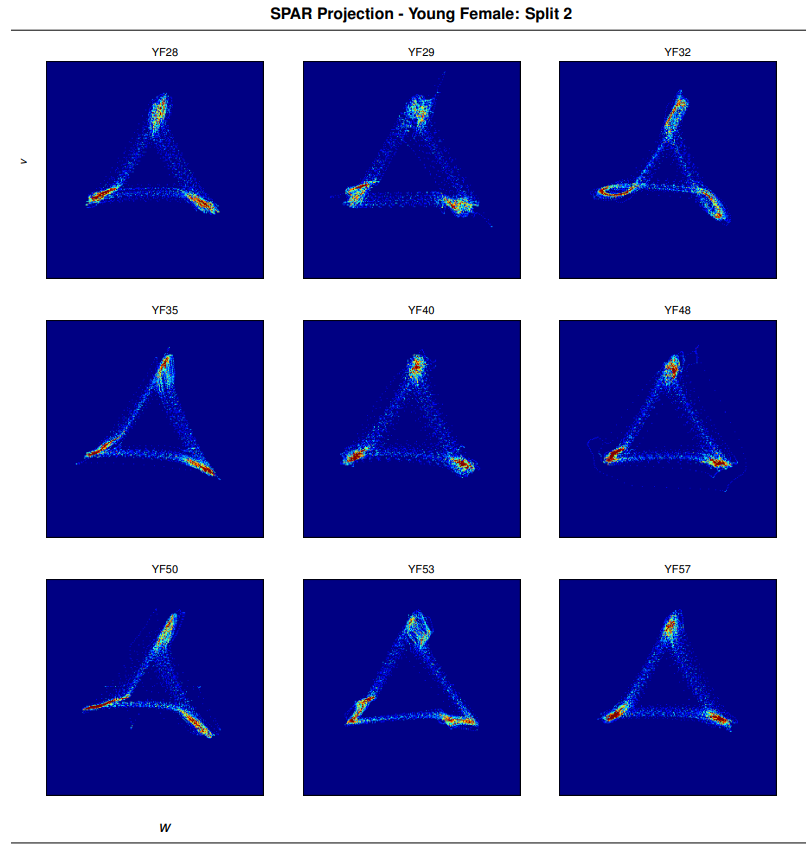
**

**Figure S10 ii** Attractor constructs of original input PPG data, taken from last 9 young female subjects of VORTAL^23^. Projections were created at 120-second window intervals and a random window per subject was selected for the figure.

**
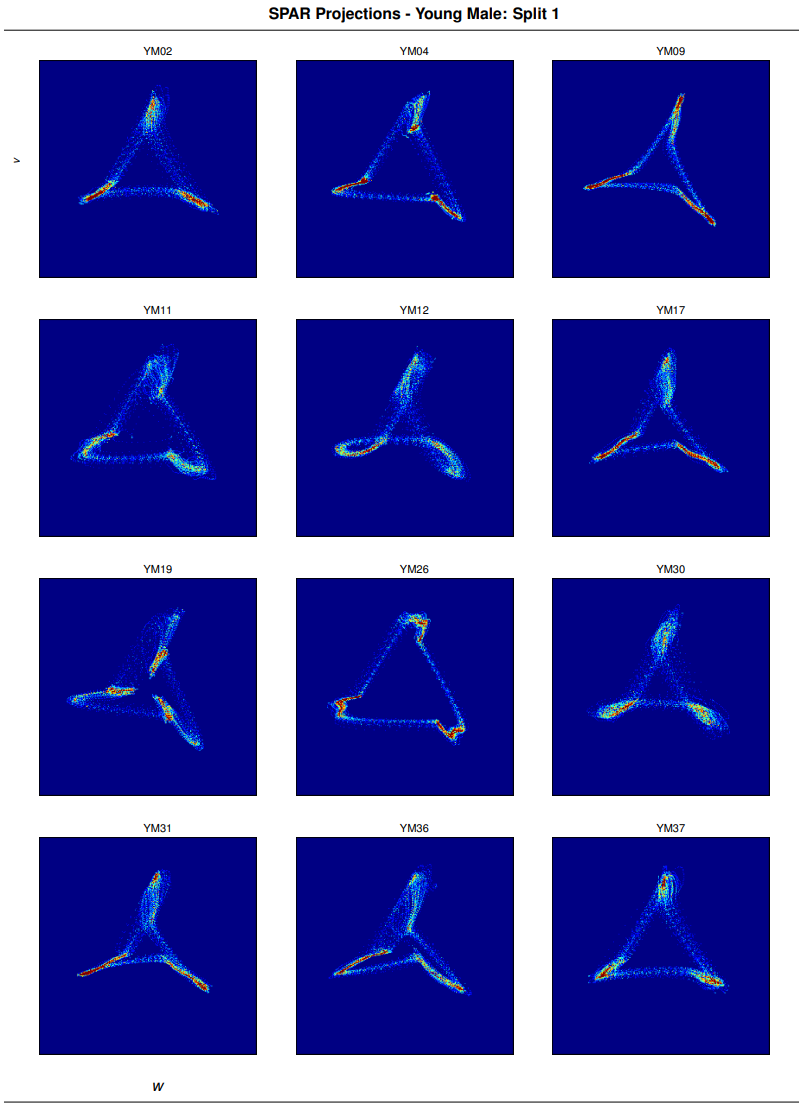
**

**Figure S11i:** Attractor constructs of original input PPG data, taken from the first 12 young male subjects of VORTAL^23^. Projections were created at 120-second window intervals and a random window per subject was selected for the figure.

**
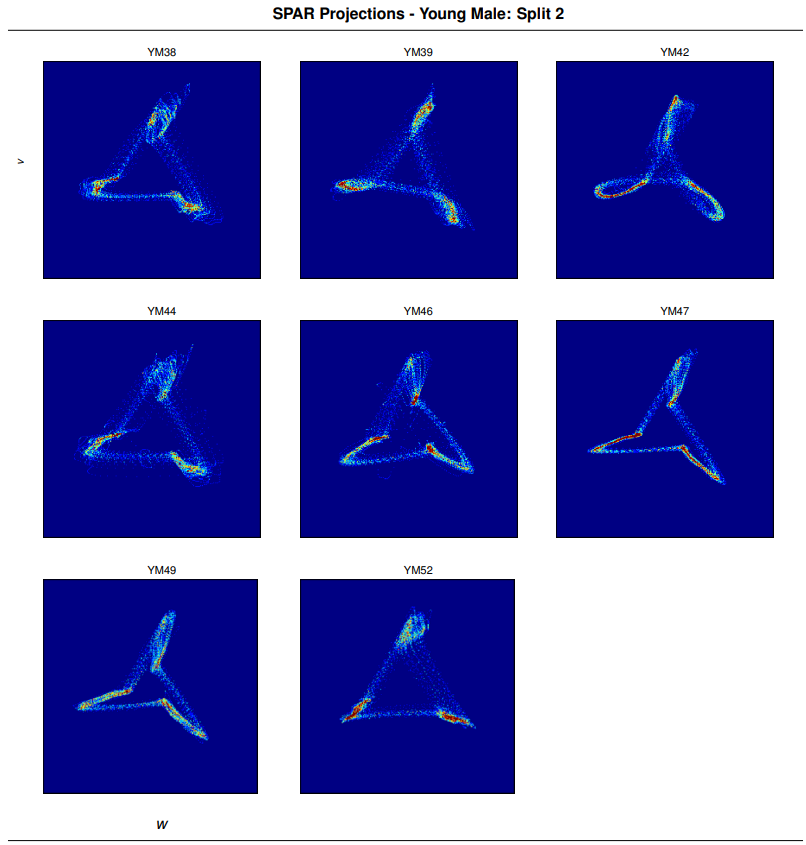
**

**Figure S11ii:** Attractor constructs of original input PPG data, taken from the last 9 young male subjects of VORTAL^23^. Projections were created at 120-second window intervals and a random window per subject was selected for the figure.
