# Supplementary material for: Advanced waveform analysis of the photoplethysmogram signal using complementary signal processing techniques for the extraction of biomarkers of cardiovascular function
Source: JRSM Cardiovasc Dis. 2024 Feb 1;13:20480040231225384. doi: 10.1177/20480040231225384 (PMC10838030; doi:10.1177/20480040231225384)
Supplement: sj-docx-2-cvd-10.1177_20480040231225384 - Supplemental material for Advanced waveform analysis of the photoplethysmogram signal using complementary signal processing techniques for the extraction of biomarkers of cardiovascular function [file sj-docx-2-cvd-10.1177_20480040231225384.docx]

| **FPA Index** | **Interpretation** |
| --- | --- |
| AI | Augmentation Index. Difference between the amplitudes of the two systolic peaks, relative to pulse pressure. |
| IPAD | Ratio of diastolic to systolic area (inflection point area) plus diastolic peak (‘dia’ in Figure 1: panel (d)). |
| c/a, d/a, e/a | Ratio of relative amplitudes between different points (a, c, d, e; see Figure 1: panel (h)) of the second derivative of the PPG waveforms. |

**Table S1:** Description of the FPA^4,9,10^ indices calculated from the PPG waveforms used in the described in-silico and in-vivo studies.
