# Supplementary material for: Advanced waveform analysis of the photoplethysmogram signal using complementary signal processing techniques for the extraction of biomarkers of cardiovascular function
Source: JRSM Cardiovasc Dis. 2024 Feb 1;13:20480040231225384. doi: 10.1177/20480040231225384 (PMC10838030; doi:10.1177/20480040231225384)
Supplement: sj-docx-3-cvd-10.1177_20480040231225384 - Supplemental material for Advanced waveform analysis of the photoplethysmogram signal using complementary signal processing techniques for the extraction of biomarkers of cardiovascular function [file sj-docx-3-cvd-10.1177_20480040231225384.docx]

| **SPAR Index** | **Interpretation** |
| --- | --- |
| Opening (n%) | Attractor **opening**, quantified by measuring the relative size of a concentric idealised circle which encompasses the n% of the attractor density data. Correlated to waveform **sinusoidality.** |
| Rotation | Attractor **rotation** compared to an idealised concentric horizontal equilateral triangle. Correlated to changes in waveform **morphology** such as wave downstroke **concavity**, amongst others. |
| Symmetry | Attractor **rotational symmetry**, calculated by duplicating and rotating the attractor figure by several angular steps and quantifying the mismatch between the original and rotated attractors. **Rounder** (more rotationally symmetrical) attractors generate lower values of this metric. Correlated to waveform **sinusoidality.** |
| Peak Width | Attractor **edge roundness**. Reduced **waveform sinusoidality** produces looped attractors with rounder corners, thus a higher value for this metric. This metric can however also be increased if attractors are perfectly round, stemming from perfectly sinusoidal waveforms. |
| Band Width | **Width** of attractor arms. Correlated to attractor and therefore wave-to-wave morphological **variability**. Higher values of this metric can identify attractors with bi-phasic (looped) arms, thus can correlate to waveform **morphology.** |
| Arm Density* | Highest density observed of an averaged attractor arm. Higher values of this metric correspond to a reduced wave-to-wave morphological **variability.** |

**Table S2:** Description of the SPAR indices calculated from the SPAR attractor constructs ^11-13^ used in the described in-silico and in-vivo studies. *Only used for the in-vivo studies, this being mainly a variability metric.
