# Supplementary material for: Advanced waveform analysis of the photoplethysmogram signal using complementary signal processing techniques for the extraction of biomarkers of cardiovascular function
Source: JRSM Cardiovasc Dis. 2024 Feb 1;13:20480040231225384. doi: 10.1177/20480040231225384 (PMC10838030; doi:10.1177/20480040231225384)
Supplement: sj-docx-4-cvd-10.1177_20480040231225384 - Supplemental material for Advanced waveform analysis of the photoplethysmogram signal using complementary signal processing techniques for the extraction of biomarkers of cardiovascular function [file sj-docx-4-cvd-10.1177_20480040231225384.docx]

|  |  |  | **HR** | |  | **SV** | |  | **LVET** | |  | **DIA** | |  | **PWV** | |  | **MAP** | |
| --- | --- | --- | --- | --- | --- | --- | --- | --- | --- | --- | --- | --- | --- | --- | --- | --- | --- | --- | --- |
|  |  |  |  |  |  |  |  |  |  |  |  |  |  |  |  |  |  |  |  |
|  |  |  | **+1SD** | **-1SD** |  | **+1SD** | **-1SD** |  | **+1SD** | **-1SD** |  | **+1SD** | **-1SD** |  | **+1SD** | **-1SD** |  | **+1SD** | **-1SD** |
|  |  |  |  |  |  |  |  |  |  |  |  |  |  |  |  |  |  |  |  |
| **FPA** | AI |  | 5 | -5 |  | -17 | 19 |  | -6 | 8 |  | 9 | 0 |  | 14 | -13 |  | 8 | -4 |
|  | IPAD |  | 32 | -27 |  | -15 | 3 |  | -2 | 0 |  | -33 | 24 |  | -8 | 10 |  | -4 | 4 |
|  | c/a |  | 50 | -20 |  | -88 | 153 |  | -17 | 31 |  | -109 | 126 |  | -14 | 65 |  | 9 | 5 |
|  | d/a |  | -26 | 55 |  | 165 | -80 |  | 34 | -25 |  | 54 | -55 |  | -5 | 20 |  | -1 | -1 |
|  | e/a |  | -9 | 5 |  | 16 | -22 |  | 3 | -4 |  | 3 | -3 |  | 11 | -18 |  | 5 | -7 |
|  |  |  |  |  |  |  |  |  |  |  |  |  |  |  |  |  |  |  |  |
| **SPAR** | Opening (5%) |  | 32 | 6 |  | -24 | 30 |  | -8 | 8 |  | -25 | 28 |  | -26 | 69 |  | -18 | 25 |
|  | Rotation |  | -18 | 24 |  | 6 | -6 |  | 0 | 0 |  | 47 | -29 |  | 29 | -24 |  | 12 | -12 |
|  | Peak Width |  | -39 | -21 |  | -24 | -18 |  | 7 | -8 |  | -15 | -32 |  | 55 | -49 |  | -34 | -26 |
|  | Band Width |  | -18 | 20 |  | 136 | 12 |  | 11 | 23 |  | 91 | 20 |  | 37 | -40 |  | 182 | 18 |
|  | Symmetry |  | -33 | 4 |  | 21 | -16 |  | 12 | -7 |  | 48 | -28 |  | 58 | -41 |  | 45 | -22 |

**Table S3:** FPA^4,9,10^ (top) and SPAR^11-13^ (bottom) indices deviations following manual changes in cardiovascular parameters on an in-silico radial PPG wave's morphology from the PWDE database'. Variations shown as percentages of change (rows) when the 25-year-old baseline PPG waveform changed was changed by +/-1SD of the different cardiovascular parameters (columns). Colour-graded by relative absolute differences. HR: heart rate; SV: stroke volume LVET: left ventricular ejection time; DIA: vessel diameter; PW: pulse wave velocity; MAP: mear arterial pressure. Refer to Table S1 for a description of the FPA indices and to Table S2 for a description of the SPAR indices.
