# Supplementary material for: Advanced waveform analysis of the photoplethysmogram signal using complementary signal processing techniques for the extraction of biomarkers of cardiovascular function
Source: JRSM Cardiovasc Dis. 2024 Feb 1;13:20480040231225384. doi: 10.1177/20480040231225384 (PMC10838030; doi:10.1177/20480040231225384)
Supplement: sj-docx-5-cvd-10.1177_20480040231225384 - Supplemental material for Advanced waveform analysis of the photoplethysmogram signal using complementary signal processing techniques for the extraction of biomarkers of cardiovascular function [file sj-docx-5-cvd-10.1177_20480040231225384.docx]

|  |  |  | **VORTAL** | | | | | | | | | | |  | **PPG Diary** | | | | |
| --- | --- | --- | --- | --- | --- | --- | --- | --- | --- | --- | --- | --- | --- | --- | --- | --- | --- | --- | --- |
|  |  |  |  |  |  |  |  |  |  |  |  |  |  |  |  |  |  |  |  |
|  |  |  | **YF-EM** |  | **YM-EM** |  | **YF-YM** |  | **EF-EM** |  | **Y-E** |  | **M-F** |  | **S-C** |  | **C-B** |  | **S-B** |
|  |  |  |  |  |  |  |  |  |  |  |  |  |  |  |  |  |  |  |  |
| **FPA** | AI |  | 0.925 |  | 0.850 |  | 0.563 |  | 0.446 |  | 0.878 |  | 0.548 |  | 0.440 |  | 0.837 |  | 0.856 |
|  | IPAD |  | 1.000 |  | 0.892 |  | 0.743 |  | 0.917 |  | 0.950 |  | 0.703 |  | 0.578 |  | 0.955 |  | 0.956 |
|  | c/a |  | 1.000 |  | 0.971 |  | 0.613 |  | 0.696 |  | 0.985 |  | 0.594 |  | 0.874 |  | 1.000 |  | 1.000 |
|  | d/a |  | 0.994 |  | 0.986 |  | 0.623 |  | 0.750 |  | 0.985 |  | 0.605 |  | 0.656 |  | 0.942 |  | 1.000 |
|  | e/a |  | 0.706 |  | 0.557 |  | 0.700 |  | 0.482 |  | 0.555 |  | 0.650 |  | 0.545 |  | 0.933 |  | 0.989 |
|  |  |  |  |  |  |  |  |  |  |  |  |  |  |  |  |  |  |  |  |
| **SPAR** | Opening (5%) |  | 0.975 |  | 0.957 |  | 0.610 |  | 0.554 |  | 0.962 |  | 0.579 |  | 0.944 |  | 0.929 |  | 1.000 |
|  | Rotation |  | 0.950 |  | 0.886 |  | 0.623 |  | 0.920 |  | 0.928 |  | 0.624 |  | 0.458 |  | 0.667 |  | 0.597 |
|  | Peak Width |  | 0.894 |  | 0.807 |  | 0.588 |  | 0.696 |  | 0.858 |  | 0.523 |  | 0.792 |  | 0.754 |  | 0.447 |
|  | Band Width |  | 0.950 |  | 0.979 |  | 0.555 |  | 0.839 |  | 0.953 |  | 0.495 |  | 0.948 |  | 0.821 |  | 1.000 |
|  | Symmetry |  | 0.663 |  | 0.661 |  | 0.618 |  | 0.804 |  | 0.649 |  | 0.636 |  | 0.771 |  | 0.746 |  | 0.622 |
|  | Arm Density |  | 0.906 |  | 0.846 |  | 0.568 |  | 0.500 |  | 0.886 |  | 0.515 |  | 0.556 |  | 0.563 |  | 0.492 |

**Table S4:** Univariate logistic regression ROC AUC performances after internal validation comparing FPA^4,9,10^ (top) and SPAR ^11-13^ (bottom) indices between different in-vivo groups. Y= Young, E = Elderly, F = Female, M = Male, S = Sleeping, C = Computer, B = Badminton. Continuously colour-graded by 0.5 to 1.0 ROC AUC performances. *Opening = 5% / 40% for VORTAL^23^ / PPG Diary^24^. Analyses performed on indices computed from 60-second waveform windows.
